# Efficacy and safety of PARP inhibitor maintenance therapy for ovarian cancer: a meta-analysis and trial sequential analysis of randomized controlled trials

**DOI:** 10.3389/fphar.2024.1460285

**Published:** 2024-09-18

**Authors:** Guojuan Sun, Yi Liu

**Affiliations:** ^1^ The Ward Section of Home Overseas Doctors, Hospital of Chengdu University of Traditional Chinese Medicine, Chengdu, China; ^2^ Department of Gynaecology and Obstetrics, Sichuan Provincial People’s Hospital, School of Medicine, University of Electronic Science and Technology of China, Chengdu, China

**Keywords:** PARP inhibitors, olaparib, niraparib, rucaparib, placebo, ovarian cancer, meta-analysis

## Abstract

**Background:**

The landscape of poly (ADP-ribose) polymerase (PARP) inhibitor treatment for ovarian cancer (OC) is continually evolving. This research aimed to evaluate the efficacy and safety of PARP inhibitors compared to placebo as a maintenance therapy for OC patients.

**Methods:**

We conducted a search of PubMed, Embase, Web of Science, and the Cochrane Library databases for randomized controlled trials (RCTs) involving the use of PARP inhibitors as maintenance therapy in OC patients, up to 16 June 2024. Data regarding progression-free survival (PFS), overall survival (OS), chemotherapy-free interval (CFI), time to first subsequent therapy or death (TFST), time to second subsequent therapy or death (TSST), and treatment-emergent adverse events (TEAEs) were aggregated. Pooled hazard ratio (HR) and their corresponding 95% confidence intervals (CI) were calculated for PFS, OS, CFI, TFST, and TSST. Additionally, the relative risk (RR) and 95% CI for TEAEs were determined.

**Results:**

This meta-analysis encompassed 20 RCTs involving 7,832 participants. The overall analysis demonstrated that maintenance therapy with PARP inhibitors led to significant improvements in PFS (HR: 0.398, 95% CI = 0.339–0.467, 95% PI = 0.219–0.724), OS (HR: 0.677, 95% CI = 0.582–0.788, 95% PI = 0.546–0.839), CFI (HR: 0.417, 95% CI = 0.368–0.472, 95% PI = 0.265–0.627), TFST (HR: 0.441, 95% CI = 0.391–0.498, 95% PI = 0.308–0.632), and TSST (HR: 0.574, 95% CI = 0.507–0.649, 95% PI = 0.488–0.674) compared with placebo. Subgroup analyses further indicated that PARP inhibitor maintenance treatment significantly improved PFS, regardless of homologous recombination status (all *p* < 0.05). However, the risks of any grade (RR = 1.046, 95% CI = 1.032–1.059, 95% PI = 1.028–1.055) and grade ≥3 TEAEs (RR = 2.931, 95% CI = 2.641–3.253, 95% PI = 2.128–3.792) were increased by PARP inhibitor maintenance therapy compared to placebo.

**Conclusion:**

Our research elucidated the benefits of maintenance therapy with PARP inhibitors in patients with OC, showing improvements in PFS, OS, CFI, TFST, and TSST. Vigilance regarding TEAEs is paramount for clinicians implementing PARP inhibitor maintenance therapy in clinical practice.

**Systematic Review Registration:**

https://www.crd.york.ac.uk/PROSPERO/, identifier CRD42024560286.

## 1 Introduction

Ovarian cancer (OC) stands as the primary cause of mortality among gynecological malignancies (Torre et al., 2018). At the time of diagnosis, roughly 75% of OC patients exhibit advanced stages of the disease (Lheureux et al., 2019; Salani et al., 2011). While early-stage OC can be effectively managed with initial platinum-based chemotherapy (CT) and standard cytoreductive surgery, the majority of patients with advanced OC (70%–80%) eventually develop resistance to platinum, leading to poor survival outcomes (Ledermann et al., 2013). Attempts to improve treatment efficacy, including intraperitoneal CT, weekly paclitaxel administration, the incorporation of bevacizumab, and BRAF (v-raf murine sarcoma viral oncogene homolog B1)/MEK (mitogen-activated protein kinase) inhibitors, have had limited success (Burger et al., 2011; Katsumata et al., 2013; Marchetti et al., 2019; Perren et al., 2011; Perrone et al., 2024). Pathogenic or likely pathogenic germline mutations in BRCA1 or BRCA2 genes are present in approximately 10%–20% of OC patients (Cancer Genome Atlas Research Network, 2011), while around 50% exhibit somatic defects in the homologous recombination repair pathway, referred to as homologous recombination deficiency (HRD) (Gupta et al., 2021; Cancer Genome Atlas Research Network, 2011). Mutations in BRCA1/2 heighten the likelihood of OC development in women. Furthermore, OC in women with germline mutations tends to be more aggressive and have a worse prognosis than those with somatic mutations, as BRCA-mutated tumors typically present with higher clinical grades and stages, and a greater potential for metastasis (Musolino et al., 2007). Research in cancer biology has underscored the significance of BRCA1/2 mutations and HRD, paving the way for targeted treatments such as poly (ADP-ribose) polymerase (PARP) inhibitors.

The suppression of PARP results in the persistence of single-strand DNA breaks, which subsequently lead to double-strand breaks necessitating repair via homologous recombination repair (HRR) (Creeden et al., 2021). In the context of pathogenic BRCA1/2 mutations or other HRD, cancer cells exhibit heightened sensitivity to PARP inhibitors due to synthetic lethality. This concurrent deficiency in both repair pathways culminates in cell death (Farmer et al., 2005). Consequently, this therapeutic approach has led to the development of a class of drugs known as PARP inhibitors. The introduction of these inhibitors has broadened the therapeutic options for OC, particularly for patients with BRCA mutations or HRD patients who are characterized by platinum sensitivity and non-BRCA mutation (Purwar et al., 2023). Presently, three PARP inhibitors have received FDA approval for OC treatment: olaparib and niraparib as monotherapies are sanctioned for maintenance therapy following primary and recurrent CT, while rucaparib is approved for maintenance in recurrent OC (Armstrong et al., 2022). Evidence suggested that olaparib, niraparib, and rucaparib are efficacious in the treatment of OC, particularly in extending progression-free survival (PFS) in patients with recurrent OC when compared to placebo (Cancanelli et al., 2022; Mengato et al., 2022; Wang et al., 2021). Additionally, evidence from previous randomized controlled trials (RCTs) indicated that PARP inhibitors markedly enhance PFS when employed as maintenance therapy in recurrent OC patients, irrespective of biomarker status such as BRCA mutation or HRD (Coleman et al., 2017; Ledermann et al., 2012; Mirza et al., 2016; Pujade-Lauraine et al., 2017). More recent RCTs have demonstrated significant improvements in PFS with PARP inhibitor maintenance therapy in newly diagnosed OC patients, regardless of the presence or absence of BRCA mutations or HRD (Banerjee et al., 2021; Coleman et al., 2019; González-Martín et al., 2019; Ray-Coquard et al., 2019).

Moreover, in a recent meta-analysis, Wang et al. demonstrated an improved prognosis for patients with newly diagnosed advanced OC undergoing PARP inhibitor maintenance therapy (Wang et al., 2020). Previous network meta-analyses have established the efficacy of olaparib, niraparib, and rucaparib in prolonging PFS in recurrent OC cases (Wang et al., 2021; Xu et al., 2020). Nonetheless, in recent years, multiple RCTs have provided updated data on PFS, overall survival (OS), chemotherapy-free interval (CFI), time to first subsequent therapy or death (TFST), and time to second subsequent therapy or death (TSST) following PARP inhibitor maintenance therapy for OC (DiSilvestro et al., 2023; González-Martín et al., 2023; Li et al., 2023; Pujade-Lauraine et al., 2023; Wu et al., 2024a; Wu et al., 2024b). Additionally, there remains debate over whether different PARP inhibitor maintenance treatments elevate the risk of any grade treatment-emergent adverse events (TEAEs) compared to placebo (Coleman et al., 2017; Friedlander et al., 2018; Monk et al., 2022). Therefore, we conducted a meta-analysis to evaluate the efficacy and safety of PARP inhibitor maintenance therapy versus placebo in the treatment of OC and its various subtypes.

## 2 Methods

### 2.1 Study protocol

This research adhered rigorously to the guidelines outlined by the Preferred Reporting Items for Systematic Reviews and Meta-Analyses (PRISMA) (Page et al., 2021). The study protocol was prospectively recorded in the PROSPERO database (CRD42024560286).

### 2.2 Search strategy

A comprehensive literature search was performed across several databases, including PubMed, Web of Science, the Cochrane Library, and Embase, to locate relevant RCTs published up to 16 June 2024. The search terms utilized included: (“poly (ADP-ribose) polymerase inhibitor,” “PARP inhibitor,” “PARPi,” “PARP inhibitors”) OR (“olaparib,” “niraparib,” “rucaparib,” “veliparib,” “AZD221,” “AG014699,” “MK 4827”) AND (“ovarian neoplasm,” “ovarian cancer,” “cancer of ovary,” “ovary cancer”). A detailed search strategy is available in Supplementary Files S1. Additionally, references within selected review articles were examined to capture further relevant studies.

### 2.3 Inclusion and exclusion criteria

The inclusion criteria for the selected articles were as follows: (1) RCTs; (2) participants were adult women (18 years and older) with a histologically or cytologically confirmed diagnosis of OC at any stage; (3) intervention involved maintenance treatment with PARP inhibitors; (4) comparison: treatment with placebo; (5) outcomes included PFS, OS, CFI, TFST, TSST, TEAEs of any grade, or grade ≥3 TEAEs. Articles were excluded if they were: (1) single-arm trials, retrospective or prospective cohort studies; (2) studies involving combination therapy of PARP inhibitors with anti-angiogenic agents or CT in the intervention group; (3) trials lacking relevant outcomes or with duplicated data; (4) conference abstracts, study protocols, case reports, and literature reviews.

### 2.4 Data extraction

Two independent reviewers undertook the screening, selection, exclusion, and data extraction phases of the study. Extracted data from each eligible study included details such as first author, publication year, trial name, study phase, disease status, sample size, median participant age, specifics of intervention and control regimens, follow-up duration, and outcomes analyzed in the meta-analysis. Primary outcomes focused on PFS and OS, while secondary outcomes encompassed CFI, TFST, TSST, and TEAEs. The CFI was defined as the interval from the final dose of prior CT to the initiation of the next CT (Ledermann et al., 2020). TFST referred to the period from randomization to the first subsequent anti-cancer treatment or death (Wu et al., 2024b), while TSST denoted the time from random assignment to the second subsequent therapy or death (DiSilvestro et al., 2023). In instances where hazard ratio (HR) data extraction was not direct, the Engauge Digitizer Version 10.8 tool and the methodology proposed by Tierney et al. were employed to derive data from Kaplan-Meier curves (Tierney et al., 2007).

### 2.5 Assessment of risk of bias

The assessment of RCTs for quality and risk of bias employed the modified Jadad scale (Jadad et al., 1996). Two independent reviewers evaluated each study based on criteria encompassing the randomization process, randomization concealment, double-blinding implementation, and the documentation of withdrawals and dropouts. Studies scoring between 0 and 3 points were deemed to be of low quality, whereas those scoring between 4 and 7 points were considered high quality.

### 2.6 Statistical analysis

The efficacy and safety outcomes are synthesized using HR and relative risk (RR), each accompanied by a 95% confidence interval (CI) and prediction interval (PI). The HR less than 1 indicated a benefit for the intervention group, while HR greater than 1 suggested an advantage for the control group. Cochran’s Q test and I^2^ statistics were used to statistically probe heterogeneity (Bowden et al., 2011; IntHout et al., 2016). When I^2^ exceeded 50% or *p*-values were below 0.10, significant heterogeneity was inferred, prompting the use of a random-effects model; otherwise, a fixed-effects model was employed (Higgins and Thompson, 2002). Subgroup analyses based on homologous recombination (HR) status, OC subtypes, or specific PARP inhibitors were performed only for groups with ≥2 studies included. Sensitivity analysis was performed to validate the stability of the current analysis. Publication bias was ascertained through the visual examination of funnel plots and application of Begg’s and Egger’s tests (Begg and Mazumdar, 1994; Egger et al., 1997), with any detected bias adjusted using the trim-and-fill method (Duval and Tweedie, 2000). All statistical analyses were conducted using R Version 4.3.1 and STATA Version 12.0, with a two-sided *p*-value of less than 0.05 considered to indicate statistical significance.

### 2.7 Trial sequential analysis

A trial sequential analysis (TSA) was executed to evaluate the robustness of the evidence and correct potential inaccuracies (Wetterslev et al., 2017). For TEAE outcomes, the TSA was conducted using TSA v0.9.5.10 Beta software to determine the required information size (RIS) and establish trial sequential monitoring boundaries. The RIS estimation and construction of O’Brien-Fleming α-spending boundaries were performed using the TSA software, maintaining a type I error at 5% and a type II error at 20%. The efficacy outcomes of PFS, OS, CFI, TFST, and TSST were analyzed using the “rsource” and “metacumbounds” functions of STATA 12.0, in conjunction with the “ldbounds” and “foreign” packages of R software 4.3.1 (Xie et al., 2022). The RIS was evaluated using an *a priori* information size (APIS) method. If the cumulative Z-curve intersected the trial sequential monitoring or RIS boundary, additional studies were deemed unnecessary, and solid evidence was gathered to either confirm or deny the effect of the intervention.

## 3 Results

### 3.1 Study selection procedure

**FIGURE 1:**
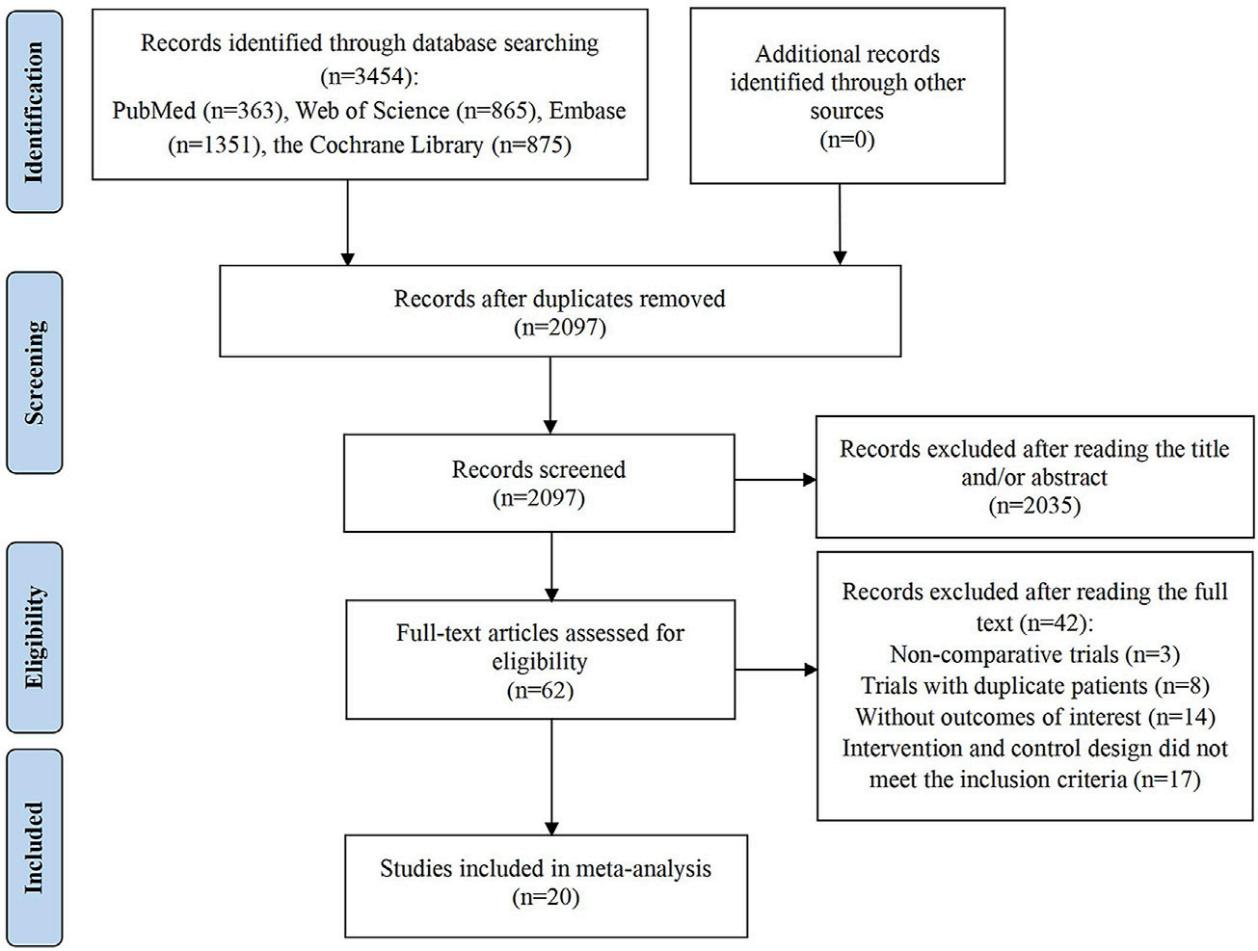
Flow diagram of the process of study selection.

The initial search yielded 3,454 articles, from which 1,357 duplicates were removed. Subsequently, title and abstract screening was performed on the remaining 2,097 articles, resulting in the exclusion of 2,035 due to irrelevance. Of the 62 full-text articles assessed, 42 were excluded for the following reasons: 3 were non-comparative clinical studies; 8 involved repeated trials; 14 lacked essential outcome data; and 17 had intervention and control designs that did not meet the inclusion criteria. Ultimately, 20 studies satisfied the inclusion criteria and were incorporated into the meta-analysis (Banerjee et al., 2021; Coleman et al., 2017; DiSilvestro et al., 2023; Friedlander et al., 2018; González-Martín et al., 2019; González-Martín et al., 2023; Ledermann et al., 2014; Ledermann et al., 2020; Li et al., 2022a; Li et al., 2023; Mirza et al., 2016; Monk et al., 2022; Moore et al., 2018; Poveda et al., 2021; Pujade-Lauraine et al., 2017; Pujade-Lauraine et al., 2023; Wu L. et al., 2021; Wu et al., 2024a; Wu et al., 2024b; Wu X. H. et al., 2021). The study identification and selection process are illustrated in Figure 1.

### 3.2 Study characteristics and quality assessment

The details of the included studies and their participants are presented in Table 1. This analysis encompassed 20 studies, comprising 2 phase II and 18 phase III trials, all published in English between 2014 and 2024. The subjects were patients with newly diagnosed, recurrent, or advanced OC. Specifically, 8 studies focused on newly diagnosed OC, 11 on recurrent OC, and 1 on advanced OC. A total of 5,204 OC patients were randomly assigned to receive maintenance therapy with PARP inhibitors, while 2,628 patients were allocated to placebo. PARP inhibitors used in the intervention group included olaparib, niraparib, rucaparib, fuzuloparib, and senaparib. Notably, only one study each reported on the efficacy and safety of fuzuloparib and senaparib as maintenance therapies for OC. All included trials were published in high-impact journals, characterized by rigorous designs and comprehensive descriptions. Consequently, all studies were considered to be of high quality. Further information on the quality assessment (Supplementary Table S1) and Risk of Bias graph (Supplementary Figure S1) are available in Supplementary Files S2.

**TABLE 1:** Characteristics of RCTs included in this meta-analysis.

First author (Year)	Trial name	Study phase	Disease state	Population (I/C)	Median age (range) (y)	Intervention arm	Control arm	Median duration of follow-up (I/C, mo)	Reported outcomes
Monk et al. (2022)	ATHENA-MONO	Phase III	Newly diagnosed, histologically confirmed, advanced, high-grade epithelial ovarian, fallopian tube, or primary peritoneal cancer; FIGO stage III-IV	427/111	I: 61 (30–83); C: 61 (31–80)	Rucaparib 600 mg twice a day	Placebo	26.1/26.2	1, 6, 7
Banerjee et al. (2021)	SOLO1/GOG 3004	Phase III	Newly diagnosed, histologically confirmed advanced, FIGO stage III or IV, high-grade serous or high-grade endometrioid ovarian cancer; ECOG-PS of 0–1	260/131	18 ears or older	Olaparib 300 mg twice daily	Placebo	57.6/60	1
Wu et al. (2021a)	NORA	Phase III	Histologically confirmed epithelial ovarian, fallopian tube or primary peritoneal carcinoma of high-grade serous histology or no histological restrictions for patients with ovarian cancer carrying a germline BRCA mutation	177/88	I: 53 (35–78); C: 55 (38–72)	Niraparib 300 mg/day	Placebo	15.8	1, 6, 7
González-Martín et al. (2019)	PRIMA/ENGOT-OV26/GOG-3012	Phase III	Newly diagnosed, histologically confirmed advanced cancer of the ovary, peritoneum, or fallopian tube; FIGO stage III or IV	487/246	I: 62 (32–85); C: 62 (33–88)	Niraparib 300 mg once daily	Placebo	13.8	2, 4
Li et al. (2022a)	FZOCUS-2	Phase III	Pathologically confirmed, high-grade (or poorly to moderately differentiated) serous ovarian cancer, primary peritoneal or fallopian tube cancer, or grade ≥2 endometrioid ovarian cancer	167/85	I: 54 (34–75); C: 54 (29–73)	Fuzuloparib 150 mg twice daily	Placebo	8.5	1, 3, 6, 7
Poveda et al. (2021)	SOLO2/ENGOT-Ov21	Phase III	Histologically confirmed, relapsed, high-grade serous or high-grade endometrioid ovarian cancer, including primary peritoneal or fallopian tube cancer; ECOG-PS of 0–1	196/99	I: 56 (IQR 51–63); C: 56 (IQR 49–63)	Olaparib 300 mg twice daily	Placebo	65.7/64.5	2, 4, 5, 6, 7
Coleman et al. (2017)	ARIEL3	Phase III	Platinum-sensitive, high-grade serous or endometrioid ovarian, primary peritoneal, or fallopian tube carcinoma	375/189	I: 61 (IQR 53–67); C: 62 (IQR 53–68)	Rucaparib 600 mg twice daily	Placebo	NR	1, 6, 7
Wu et al. (2021b)	SOLO1 (China cohort)	Phase III	Newly diagnosed, histologically confirmed advanced high-grade serous ovarian cancer or high-grade endometrioid cancer	44/20	18 years or older	Olaparib 300 mg twice daily	Placebo	30.5/30.4	1, 4, 5, 6, 7
Friedlander et al. (2018)	Study 19	Phase II	Recurrent, platinum-sensitive, ovarian, fallopian tube or primary peritoneal cancer with high-grade serous histology	136/129	I: 58 (21–89); C: 59 (33–84)	Olaparib 400 mg twice daily	Placebo	78	2, 4, 5, 6, 7
Ledermann et al. (2014)	Study 19	Phase II	Recurrent, platinum-sensitive, ovarian or fallopian tube cancer, or primary peritoneal cancer, with high-grade (grade 2 or 3) serous features or a serous component	136/129	I: 58 (21–89); C: 59 (33–84)	Olaparib 400 mg twice daily	Placebo	5.6	1
Mirza et al. (2016)	ENGOT-OV16/NOVA	Phase III	Histologically diagnosed ovarian cancer, fallopian tube cancer, or primary peritoneal cancer with predominantly high-grade serous histologic features	372/181	I: NR (33–84); C: NR (34–82)	Niraparib 300 mg once daily	Placebo	16.9	1, 3, 4, 6, 7
Moore et al. (2018)	SOLO1	Phase III	Newly diagnosed, histologically confirmed advanced (FIGO stage III or IV) high-grade serous or endometrioid ovarian cancer, primary peritoneal cancer, or fallopian-tube cancer	260/131	18 years or older	Olaparib 300 mg twice daily	Placebo	40.7/41.2	1, 6
Pujade-Lauraine et al. (2017)	SOLO2/ENGOT-Ov21	Phase III	Histologically confirmed, relapsed, high-grade serous ovarian cancer or high-grade endometrioid cancer; ECOG-PS of 0–1	196/99	I: 56 (IQR 51–63); C: 56 (IQR 49–63)	Olaparib 300 mg twice daily	Placebo	22.1/22.2	1
Wu et al. (2024a)	FLAMES	Phase III	Histologically confirmed advanced (FIGO stage III-IV), high-grade serous or endometrioid cancer or other histological types of epithelial ovarian cancer, fallopian tube cancer or primary peritoneal cancer; ECOG-PS of 0–1	271/133	I: 55 (IQR 50–62); C: 54 (IQR 49–60)	Senaparib 100 mg once daily	Placebo	22.3	1, 3, 4, 6, 7
Li et al. (2023)	PRIME	Phase III	New diagnosis of histologically confirmed, high-grade serous or endometrioid epithelial ovarian cancer, fallopian tube carcinoma, or primary peritoneal carcinoma; FIGO stage III or IV	255/129	I: 53 (32–77); C: 54 (33–77)	Niraparib 200 mg or 300 mg once daily	Placebo	27.5	1, 2, 4, 6, 7
González-Martín et al. (2023)	PRIMA/ENGOT-OV26/GOG-3012	Phase III	Newly diagnosed, advanced (FIGO stage III/IV), high-grade serous or endometrioid ovarian, primary peritoneal, or fallopian tube cancer	487/246	I: 62 (32–85); C: 62 (33–88)	Niraparib 300 mg once daily	Placebo	41.6/41.9	1, 6, 7
DiSilvestro et al. (2023)	SOLO1/GOG 3004	Phase III	Newly diagnosed, histologically confirmed advanced (FIGO stage III or IV) high-grade serous or endometrioid ovarian, primary peritoneal, and/or fallopian tube cancer	260/131	18 years or older	Olaparib 300 mg twice daily	Placebo	88.9/87.4	2, 4, 5, 7
Wu et al. (2024b)	NORA	Phase III	Histologically confirmed, recurrent, (predominantly) high-grade serous epithelial ovarian cancer, fallopian tube carcinoma, or primary peritoneal carcinoma; ECOG-PS of 0 or 1	177/88	I: 53 (35–78); C: 55 (38–72)	Niraparib 300 mg/day	Placebo	58.4/57.0	2, 3, 4
Pujade-Lauraine et al. (2023)	OReO/ENGOT-ov38	Phase III	Relapsed histologically diagnosed non-mucinous epithelial ovarian cancer, primary peritoneal cancer, and/or fallopian tube cancer	146/74	I: NR (29–81); C: NR (43–87)	Olaparib 300 mg twice daily	Placebo	Cohort 1: 4.1/2.8; Cohort 2: 2.9/2.8	1, 2, 4, 5, 6, 7
Ledermann et al. (2020)	ARIEL3	Phase III	Platinum-sensitive, high-grade serous or endometrioid ovarian, primary peritoneal, or fallopian tube carcinoma; ECOG-PS of 0 or 1	375/189	I: 61 (IQR 53–67); C: 62 (IQR 53–68)	Rucaparib 600 mg twice daily	Placebo	28.1	3, 4, 5

I, intervention; C, control; y, year; mo, month; FIGO, international federation of gynecology and obstetrics; ECOG-PS, eastern cooperative oncology group performance status; IQR, interquartile range; NR, not reported; 1, progression-free survival; 2, overall survival; 3, chemotherapy-free interval; 4, time to first subsequent therapy or death; 5, time to second subsequent therapy or death; 6, any grade treatment-emergent adverse events (TEAEs); 7, grade ≥3 TEAEs.

### 3.3 Pooled effect of primary outcomes

**FIGURE 2:**
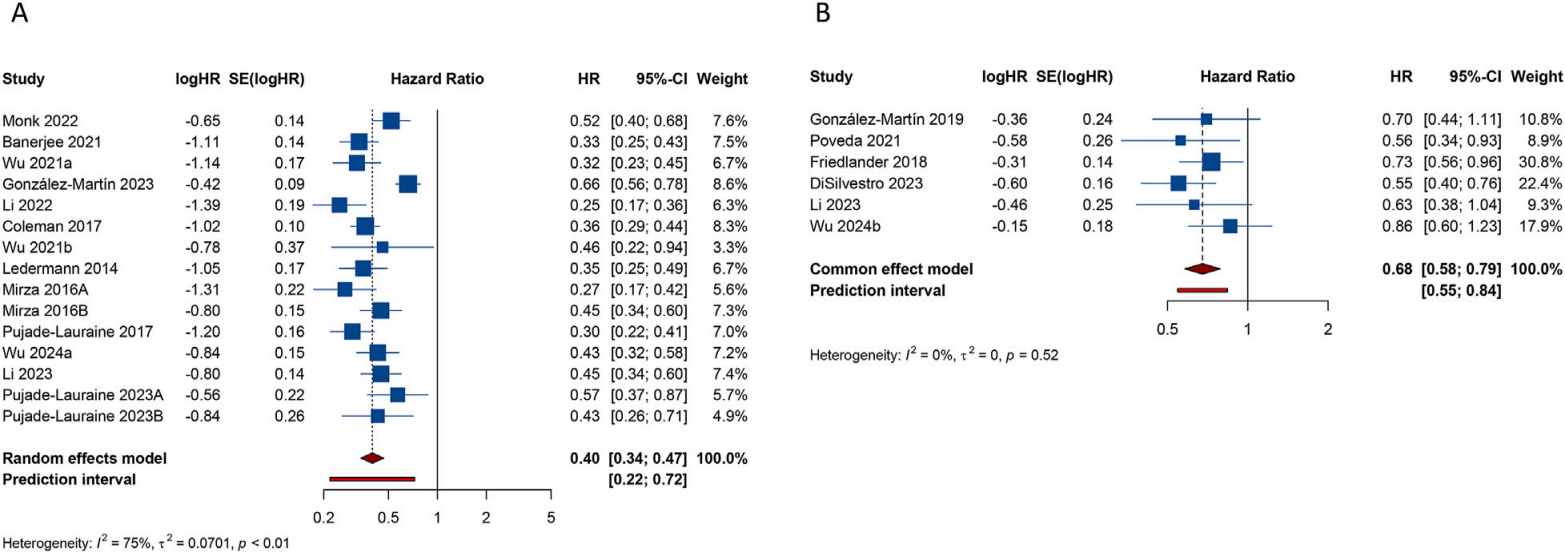
Forest plot of primary outcomes after PARP inhibitor maintenance therapy for ovarian cancer. **(A)** progression-free survival; **(B)** overall survival.

Fifteen studies investigated the PFS benefit of PARP inhibitors in OC patients. A pooled analysis using random-effects model (I^2^ = 75.0%, Tau^2^ = 0.0701) indicated a 60.2% reduction in the risk of disease progression or mortality with PARP inhibitor maintenance therapy compared to placebo (HR: 0.398, 95% CI = 0.339–0.467, 95% PI = 0.219–0.724) (Table 2; Figure 2A). Subgroup analyses based on HR status demonstrated significant PFS improvements across various HR categories, including HRD (HR: 0.427, 95% CI = 0.368–0.496, 95% PI = 0.232–0.782), BRCA mutation (HR: 0.341, 95% CI = 0.269–0.432, 95% PI = 0.166–0.699), germline BRCA mutation (HR: 0.256, 95% CI = 0.203–0.323, 95% PI = 0.120–0.530), non-germline BRCA mutation (HR: 0.450, 95% CI = 0.376–0.540, 95% PI = 0.303–0.670), BRCA wild-type (HR: 0.523, 95% CI = 0.442–0.620, 95% PI = 0.412–0.665), or HR proficiency (HRP) (HR: 0.615, 95% CI = 0.497–0.761, 95% PI = 0.154–2.452). Notably, PARP inhibitors conferred PFS benefits in both newly diagnosed (HR: 0.479, 95% CI = 0.362–0.633, 95% PI = 0.180–1.273) and recurrent OC cases (HR: 0.354, 95% CI = 0.318–0.395, 95% PI = 0.238–0.524). Analysis by specific PARP inhibitors showed that olaparib (HR: 0.363, 95% CI = 0.312–0.422, 95% PI = 0.240–0.576), niraparib (HR: 0.422, 95% CI = 0.306–0.582, 95% PI = 0.130–1.370), or rucaparib (HR: 0.428, 95% CI = 0.299–0.614) maintenance therapy significantly improved PFS compared with placebo (Table 3; Supplementary Figures S2–S4).

**TABLE 2:** Pooled effect of the efficacy and safety of PARP inhibitor maintenance treatment for ovarian cancer.

Outcomes	Number of studies	Meta-analysis				Heterogeneity	
		HR/RR	95% CI	*p*-value	95% PI	I^2^, Tau^2^	*p*-value
PFS	15	0.398	0.339–0.467	<0.001	0.219–0.724	75.0%, 0.0701	<0.001
OS	6	0.677	0.582–0.788	<0.001	0.546–0.839	0%, 0	0.515
CFI	6	0.417	0.368–0.472	<0.001	0.265–0.627	39.3%, 0.0167	0.144
TFST	13	0.441	0.391–0.498	<0.001	0.308–0.632	50.4%, 0.0229	0.019
TSST	7	0.574	0.507–0.649	<0.001	0.488–0.674	0%, 0	0.579
TEAEs of any grade	13	1.046	1.032–1.059	<0.001	1.028–1.055	0%, 0	0.957
Grade ≥3 TEAEs	13	2.931	2.641–3.253	<0.001	2.128–3.792	25.7%, 0.0131	0.185

PFS, progression-free survival; OS, overall survival; CFI, chemotherapy-free interval; TFST, time to first subsequent therapy or death; TSST, time to second subsequent therapy or death; TEAEs, treatment-emergent adverse events.

**TABLE 3:** Subgroup analysis of the efficacy and safety of PARP inhibitor maintenance treatment for ovarian cancer.

Outcomes and subgroups	Number of studies	Meta-analysis				Heterogeneity	
		HR/RR	95% CI	*p*-value	95% PI	I^2^, Tau^2^	*p*-value
PFS							
Homologous recombination status							
HRD	5	0.427	0.368–0.496	<0.001	0.232–0.782	45.0%, 0.0250	0.122
BRCA mutation	9	0.341	0.269–0.432	<0.001	0.166–0.699	62.3%, 0.0775	0.007
Germline BRCA mutation	5	0.256	0.203–0.323	<0.001	0.120–0.530	31.5%, 0.0331	0.212
Non-germline BRCA mutation	4	0.450	0.376–0.540	<0.001	0.303–0.670	0%, 0	0.932
BRCA wild-type	6	0.523	0.442–0.620	<0.001	0.412–0.665	0%, 0	0.620
HRP	3	0.615	0.497–0.761	<0.001	0.154–2.452	0%, 0	0.386
OC subtypes							
Newly diagnosed OC	5	0.479	0.362–0.633	<0.001	0.180–1.273	79.3%, 0.0741	0.001
Recurrent OC	9	0.354	0.318–0.395	<0.001	0.238–0.524	43.5%, 0.0220	0.078
Types of PARP inhibitors							
Olaparib vs. Placebo	6	0.363	0.312–0.422	<0.001	0.240–0.576	29.5%, 0.0157	0.214
Niraparib vs. Placebo	5	0.422	0.306–0.582	<0.001	0.130–1.370	84.2%, 0.1099	<0.001
Rucaparib vs. Placebo	2	0.428	0.299–0.614	<0.001	—	78.5%, 0.0531	0.031
OS							
Homologous recombination status							
HRD	2	0.752	0.440–1.286	0.298	—	0%, 0	0.508
BRCA mutation	2	0.701	0.509–0.966	0.030	—	4.7%, 0.0029	0.306
Germline BRCA mutation	2	0.738	0.559–0.975	0.033	—	0%, 0	0.587
OC subtypes							
Newly diagnosed OC	3	0.602	0.477–0.761	<0.001	0.133–2.730	0%, 0	0.689
Recurrent OC	3	0.737	0.604–0.901	0.003	0.202–2.696	0%, 0	0.400
Types of PARP inhibitors							
Olaparib vs. Placebo	3	0.635	0.524–0.770	<0.001	0.181–2.225	0.2%, <0.0001	0.367
Niraparib vs. Placebo	3	0.752	0.588–0.962	0.023	0.152–3.716	0%, 0	0.573
CFI							
OC subtypes							
Recurrent OC	5	0.402	0.326–0.497	<0.001	0.213–0.760	51.4%, 0.0283	0.084
Types of PARP inhibitors							
Niraparib vs. Placebo	3	0.407	0.286–0.581	<0.001	0.007–22.336	68.3%, 0.0666	0.043
TFST							
Homologous recombination status							
HRD	2	0.416	0.338–0.512	<0.001	—	0%, 0	0.446
BRCA mutation	3	0.366	0.247–0.543	<0.001	0.005–29.785	65.5%, 0.0794	0.055
OC subtypes							
Newly diagnosed OC	4	0.492	0.364–0.664	<0.001	0.139–1.742	73.2%, 0.0630	0.011
Recurrent OC	8	0.419	0.378–0.465	<0.001	0.329–0.531	19.5%, 0.0058	0.275
Types of PARP inhibitors							
Olaparib vs. Placebo	6	0.399	0.347–0.458	<0.001	0.327–0.486	0%, 0	0.567
Niraparib vs. Placebo	5	0.468	0.367–0.598	<0.001	0.201–1.092	72.6%, 0.0553	0.006
TSST							
Homologous recombination status							
BRCA mutation	3	0.529	0.416–0.673	<0.001	0.060–4.694	19.2%, 0.0108	0.290
OC subtypes							
Newly diagnosed OC	2	0.506	0.383–0.668	<0.001	—	0%, 0	0.828
Recurrent OC	5	0.591	0.515–0.679	<0.001	0.473–0.740	0%, 0	0.447
Types of PARP inhibitors							
Olaparib vs. Placebo	6	0.534	0.461–0.619	<0.001	0.433–0.658	0%, 0	0.895
TEAEs of any grade							
OC subtypes							
Newly diagnosed OC	5	1.054	1.032–1.078	<0.001	1.018–1.092	0%, 0	0.968
Recurrent OC	7	1.043	1.025–1.062	<0.001	1.021–1.065	0%, 0	0.999
Types of PARP inhibitors							
Olaparib vs. Placebo	5	1.049	1.018–1.081	0.002	1.004–1.098	0%, 0	0.955
Niraparib vs. Placebo	4	1.053	1.033–1.073	<0.001	1.009–1.095	0%, 0	0.973
Rucaparib vs. Placebo	2	1.041	1.012–1.071	0.005	-	0%, 0	0.850
Grade ≥3 TEAEs							
OC subtypes							
Newly diagnosed OC	5	2.771	2.374–3.235	<0.001	1.614–4.437	30.4%, 0.0152	0.219
Recurrent OC	7	3.026	2.592–3.533	<0.001	1.757–4.802	37.1%, 0.0272	0.145
Types of PARP inhibitors							
Olaparib vs. Placebo	5	2.120	1.715–2.620	<0.001	1.491–2.954	0%, 0	0.927
Niraparib vs. Placebo	4	3.107	2.666–3.621	<0.001	2.221–4.349	0%, 0	0.886
Rucaparib vs. Placebo	2	3.208	2.500–4.115	<0.001	-	48.4%, 0.0305	0.164

PFS, progression-free survival; HRD, homologous recombination deficiency; HRP, homologous recombination proficiency; OC, ovarian cancer; OS, overall survival; CFI, chemotherapy-free interval; TFST, time to first subsequent therapy or death; TSST, time to second subsequent therapy or death; TEAEs, treatment-emergent adverse events.

Six studies evaluated OS benefits. These trials exhibited no significant heterogeneity, thus adopting a fixed-effects model for analysis (I^2^ = 0%, Tau^2^ = 0). Overall, PARP inhibitor maintenance therapy significantly improved OS in OC patients relative to placebo (HR = 0.677, 95% CI = 0.582–0.788; 95% PI = 0.546–0.839) (Table 2; Figure 2B). Stratified analysis by HR status revealed improved OS in OC patients with BRCA mutation (HR = 0.701, 95% CI = 0.509–0.966) or germline BRCA mutation (HR = 0.738, 95% CI = 0.559–0.975). Furthermore, subgroup analyses by OC subtypes revealed an improved OS in patients with newly diagnosed OC (HR: 0.602, 95% CI = 0.477–0.761, 95% PI = 0.133–2.730) or recurrent OC (HR: 0.737, 95% CI = 0.604–0.901, 95% PI = 0.202–2.696). Analysis by specific PARP inhibitors suggested that olaparib (HR: 0.635, 95% CI = 0.524–0.770, 95% PI = 0.181–2.225) or niraparib (HR: 0.752, 95% CI = 0.588–0.962, 95% PI = 0.152–3.716) maintenance therapy significantly improved OS for OC patients (Table 3; Supplementary Figures S5–S7).

### 3.4 Pooled effect of secondary outcomes

#### 3.4.1 CFI, TFST, and TSST

**FIGURE 3:**
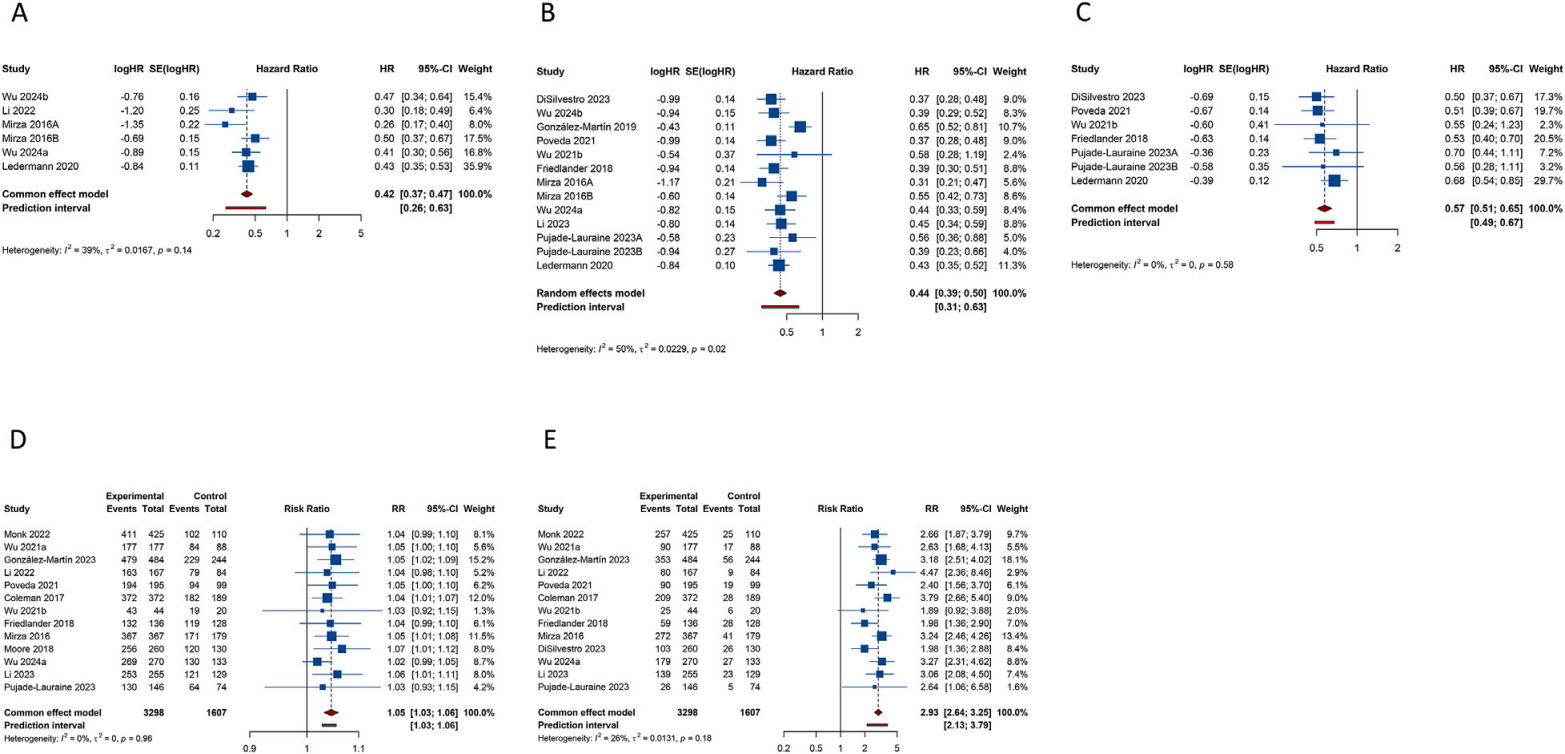
Forest plot of secondary outcomes after PARP inhibitor maintenance therapy for ovarian cancer. **(A)** Chemotherapy-free interval; **(B)** Time to first subsequent therapy or death; **(C)** Time to second subsequent therapy or death; **(D)** Any grade treatment-emergent adverse events (TEAEs); **(E)** Grade ≥3 TEAEs.

Six studies reported on the clinical benefit of CFI. The aggregated data indicated that PARP inhibitor maintenance therapy significantly prolonged CFI compared to placebo (HR: 0.417, 95% CI = 0.368–0.472, 95% PI = 0.265–0.627) (Table 2; Figure 3A). Subgroup analyses, stratified by OC subtypes or specific PARP inhibitors, demonstrated that this maintenance therapy notably prolonged CFI in recurrent OC patients (HR: 0.402, 95% CI = 0.326–0.497, 95% PI = 0.213–0.760), with niraparib showing a longer CFI than placebo (HR: 0.407, 95% CI = 0.286–0.581, 95% PI = 0.007–22.336) (Table 3; Supplementary Figure S8).

Thirteen studies examined the TFST outcome. The pooled results revealed that maintenance therapy with PARP inhibitors significantly lengthened TFST relative to placebo (HR: 0.441, 95% CI = 0.391–0.498, 95% PI = 0.308–0.632) (Table 2; Figure 3B), with consistent findings across OC patients with HRD (HR: 0.416, 95% CI = 0.338–0.512), BRCA mutation (HR: 0.366, 95% CI = 0.247–0.543, 95% PI = 0.005–29.785), and in both newly diagnosed (HR: 0.492, 95% CI = 0.364–0.664, 95% PI = 0.139–1.742) and recurrent OC (HR: 0.419, 95% CI = 0.378–0.465, 95% PI = 0.329–0.531) patients. Subsequent analysis grouped by specific PARP inhibitors suggested that olaparib (HR: 0.399, 95% CI = 0.347–0.458, 95% PI = 0.327–0.486) or niraparib (HR: 0.468, 95% CI = 0.367–0.598, 95% PI = 0.201–1.092) maintenance therapy significantly prolonged TFST compared with placebo (Table 3; Supplementary Figures S9–S11).

The TSST was evaluated in 7 studies, with combined estimates showing that PARP inhibitor maintenance therapy significantly extended TSST over placebo (HR: 0.574, 95% CI = 0.507–0.649, 95% PI = 0.488–0.674) (Table 2; Figure 3C). Subgroup analyses further indicated that this therapeutic approach substantially prolonged TSST in patients with BRCA mutation (HR: 0.529, 95% CI = 0.416 to 0.673, 95% PI = 0.060–4.694), and in both newly diagnosed (HR: 0.506, 95% CI = 0.383–0.668) and recurrent OC (HR: 0.591, 95% CI = 0.515–0.679, 95% PI = 0.473–0.740) patients. When stratified by specific PARP inhibitors, olaparib maintenance therapy was associated with a notably longer TSST compared to placebo (HR: 0.534, 95% CI = 0.461 to 0.619, 95% PI = 0.433–0.658) (Table 3; Supplementary Figure S12).

#### 3.4.2 TEAEs

Thirteen studies provided data on any grade TEAEs. The overall analysis revealed that PARP inhibitor maintenance therapy was associated with a higher risk of any grade TEAEs compared to placebo (RR = 1.046, 95% CI = 1.032–1.059, 95% PI = 1.028–1.055) (Table 2; Figure 3D). When categorized by OC subtypes, it was observed that PARP inhibitor maintenance treatment significantly increased the risk of any grade TEAEs in patients with newly diagnosed (RR = 1.054, 95% CI = 1.032–1.078, 95% PI = 1.018–1.092) or recurrent OC (RR = 1.043, 95% CI = 1.025–1.062, 95% PI = 1.021–1.065). Subgroup analyses based on specific PARP inhibitors suggested that olaparib (RR = 1.049, 95% CI = 1.018–1.081, 95% PI = 1.004–1.098), niraparib (RR = 1.053, 95% CI = 1.033–1.073, 95% PI = 1.009–1.095), or rucaparib (RR = 1.041, 95% CI = 1.012–1.071) maintenance treatment significantly increased the incidence of any grade TEAEs compared with placebo (Table 3; Supplementary Figures S13, S14).

Thirteen studies reported on grade ≥3 TEAEs. The overall findings suggested that PARP inhibitor maintenance therapy significantly elevated the risk of grade ≥3 TEAEs compared to placebo (RR = 2.931, 95% CI = 2.641–3.253, 95% PI = 2.128–3.792) (Table 2; Figure 3E). Similar results were also obtained in newly diagnosed (RR = 2.771, 95% CI = 2.374–3.235, 95% PI = 1.614–4.437) or recurrent OC (RR = 3.026, 95% CI = 2.592–3.533, 95% PI = 1.757–4.802) cases. Subgroup analysis according to the types of PARP inhibitors showed that maintenance treatment with olaparib (RR = 2.120, 95% CI = 1.715–2.620, 95% PI = 1.491–2.954), niraparib (RR = 3.107, 95% CI = 2.666–3.621, 95% PI = 2.221–4.349), or rucaparib (RR = 3.208, 95% CI = 2.500–4.115) significantly increased the incidence of grade ≥3 TEAEs compared to placebo (Table 3; Supplementary Figures S15, S16).

### 3.5 TSA results

**FIGURE 4:**
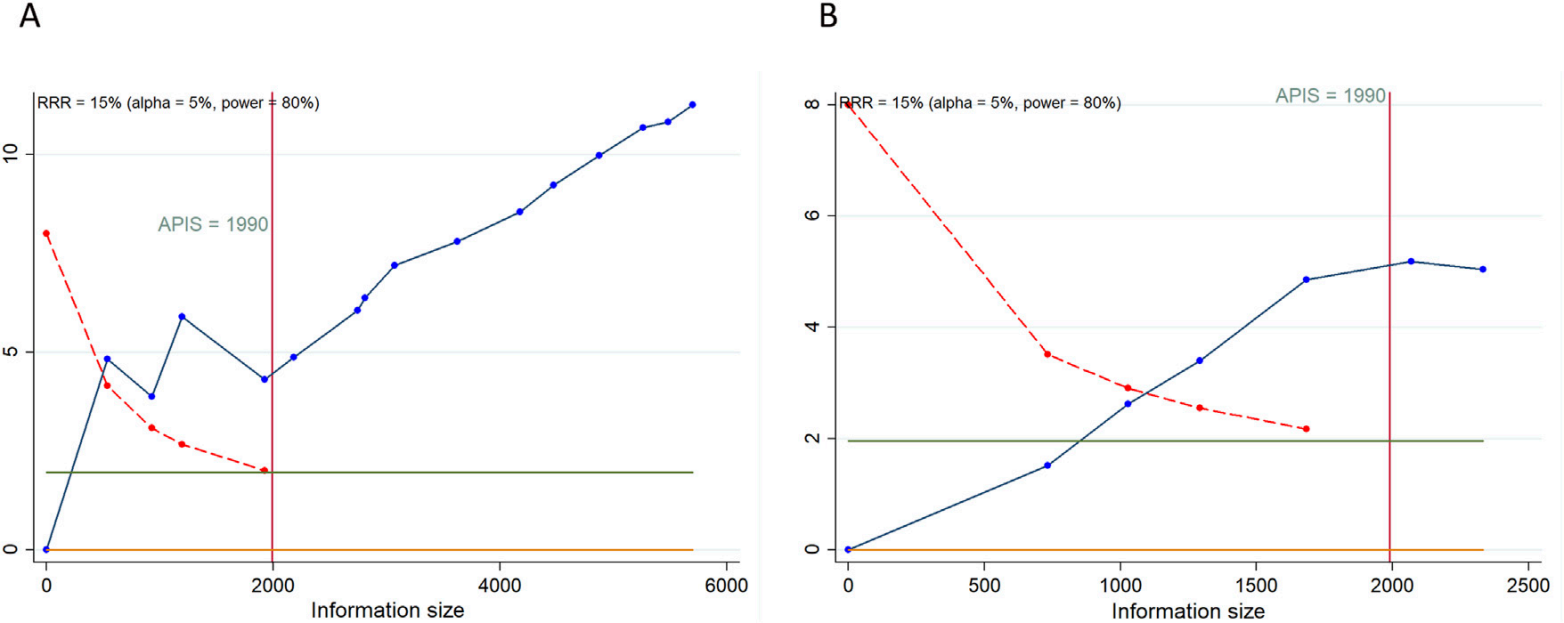
Trial sequential analysis of primary outcomes after PARP inhibitor maintenance therapy for ovarian cancer. **(A)** progression-free survival; **(B)** overall survival. Red inward-sloping line to the left represents trial sequential monitoring boundary. Blue line represents evolution of cumulative Z-score. Horizontal green lines represent the conventional boundaries for statistical significance. Heterogeneity-adjusted required information size to demonstrate or reject 15% relative risk (*a priori* estimate) of mortality risk (with alpha of 5% and beta of 20%) is 1990 patients for PFS and OS (vertical red line). Cumulative Z-curve crossing the trial sequential monitoring boundary or the APIS boundary provides firm evidence of effect.

**FIGURE 5:**
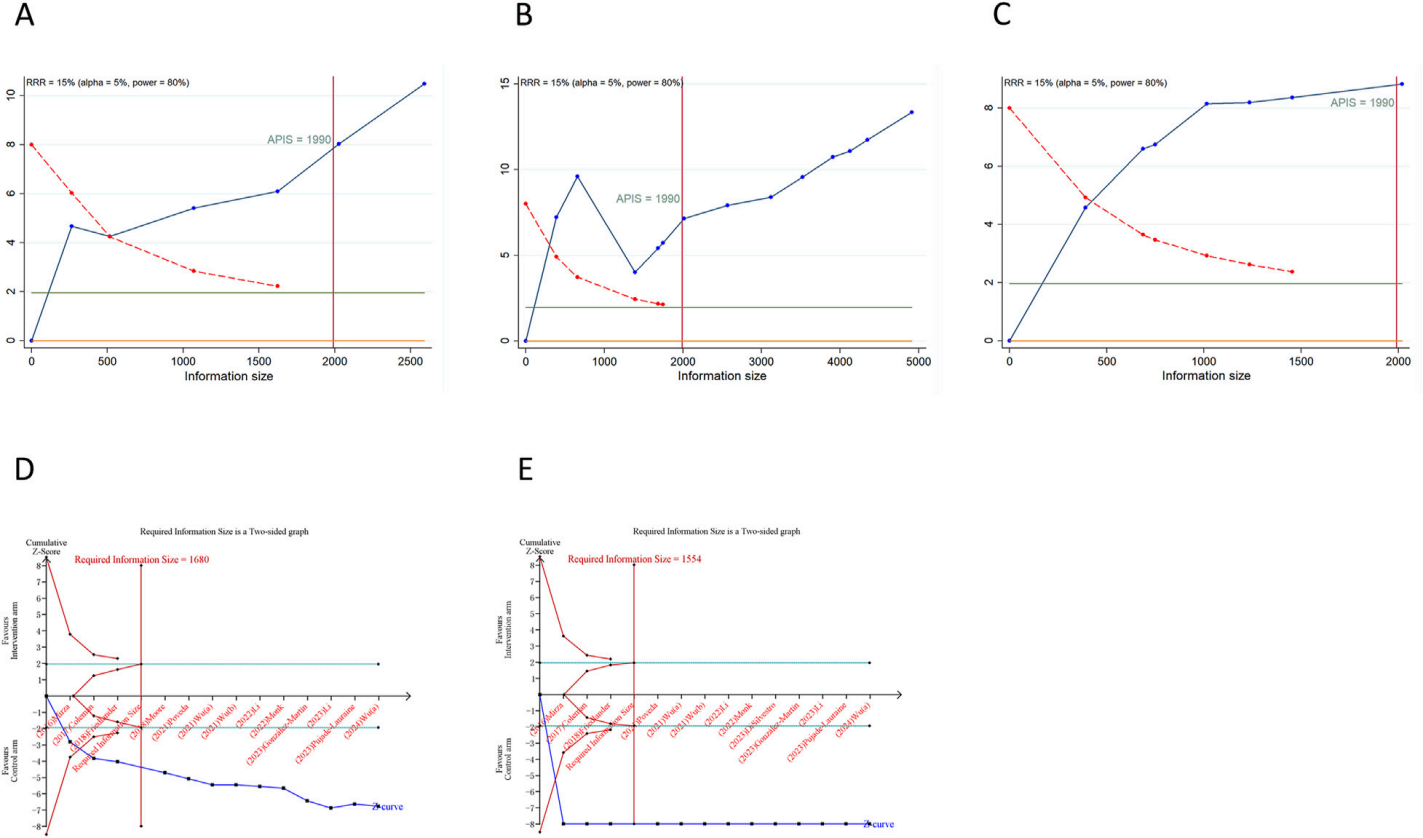
Trial sequential analysis of secondary outcomes after PARP inhibitor maintenance therapy for ovarian cancer. **(A)** Chemotherapy-free interval; **(B)** Time to first subsequent therapy or death; **(C)** Time to second subsequent therapy or death; **(D)** Any grade treatment-emergent adverse events (TEAEs); **(E)** Grade ≥3 TEAEs. Uppermost and lowermost red curves represent trial sequential monitoring boundary lines for benefit and harm, respectively. Inner red lines represent the futility boundary. Blue line represents evolution of cumulative Z-score. Horizontal green lines represent the conventional boundaries for statistical significance. Cumulative Z-curve crossing the trial sequential monitoring boundary or the RIS boundary provides firm evidence of effect.

As depicted in Figures 4, 5, a RIS of 1,990 was determined for PFS, OS, CFI, TFST, and TSST. The analysis revealed that all cumulative Z-curves surpassed both the RIS and trial sequential monitoring boundaries, indicating the attainment of a relatively definitive conclusion. For TEAEs, we determined a RIS of 1,680 for any grade TEAEs and 1,554 for grade ≥3 TEAEs. Notably, each cumulative Z-curve crossed either the RIS or trial sequential monitoring boundary, implying that additional research may not be necessary to achieve a conclusive result.

### 3.6 Sensitivity analysis and publication bias

During the sensitivity analysis, pooled HR or RR along with their 95% CI were calculated, omitting individual studies one by one to assess the influence of each study on the overall outcomes. This analysis indicated that excluding any single study did not notably alter the quantitative results, suggesting that the combined findings are robust and reliable (Supplementary Figures S17, S18). To assess publication bias, Begg’s and Egger’s tests were utilized, revealing no significant publication bias across all efficacy and safety outcomes (all *p* > 0.05). Detailed funnel plots can be found in Supplementary Figures S19, S20.

## 4 Discussion

Our meta-analysis comprehensively assessed the efficacy and safety of PARP inhibitor maintenance monotherapy compared with placebo in the treatment of OC by incorporating the outcomes of the latest RCTs. The findings indicated that PARP inhibitor maintenance therapy significantly improved PFS and OS, as well as prolonged CFI, TFST, and TSST in OC patients. Recent systematic reviews and meta-analyses mainly focused on elucidating the effects and toxicity of PARP inhibitor therapy for patients with various subtypes of OC, such as newly diagnosed, recurrent, or advanced cases (Gulia et al., 2022; Maiorano et al., 2022; Wang et al., 2021). Baradács et al.’s summary analysis demonstrated significant PFS benefits with PARP inhibitor maintenance therapy versus placebo in recurrent OC across the entire cohort, BRCA mutation carriers, germline BRCA mutation carriers, and those with wild-type BRCA status. In newly diagnosed OC, PFS was also improved in both the overall population and the BRCA mutation subgroup (Baradács et al., 2024). However, due to immature OS data in the original trials, Baradács et al.’s study has not yet confirmed the OS benefit of PARP inhibitor maintenance therapy. Additionally, Lee et al.’s research confirmed superior PFS in patients with newly diagnosed advanced epithelial OC treated with PARP inhibitors compared to placebo. Moreover, patients with HRD, BRCA wild type, BRCA1/2 mutation, or HRD without BRCA mutation, but not HRP, exhibited significantly better PFS in the PARP inhibitor group than in the placebo group. Patients with BRCA mutation in the PARP inhibitor group also had significantly better OS compared to those in the placebo group (Lee et al., 2023). Our subgroup analysis demonstrated that compared with placebo, PARP inhibitor maintenance therapy significantly improved PFS in patients with HRD, BRCA mutation, germline BRCA mutation, non-germline BRCA mutation, BRCA wild-type, or HRP. The combined analysis of mature OS data further indicated a notable improvement in OS for patients with BRCA mutation or those with germline BRCA mutation, under PARP inhibitor maintenance therapy. Furthermore, in cases of either newly diagnosed or recurrent OC, the utilization of PARP inhibitors as maintenance therapy has demonstrated significant improvements in both PFS and OS.

The mechanism by which PARP inhibitors operate in treating OC has been extensively researched. As previously noted, PARP plays a pivotal role in DNA single-strand break repair (SSBR). Inhibition of PARP can result in deficiencies in both SSBR and HRD in patients with BRCA1/2 mutations, ultimately causing cell death (Farmer et al., 2005). Homologous recombination represents a vital error-free mechanism for repairing double-strand breaks (DSBs) during cell division, necessitating functional BRCA1/2 proteins. Mutations in BRCA1/2 genes impede the homologous recombination process. Moreover, PARP inhibitors can partially impede the PARP-associated homologous recombination pathway (Lau et al., 2022). While the absence of either an operational base excision repair pathway or homologous recombination alone does not affect cell viability, the concurrent deficiency of both can result in synthetic lethality (Walsh, 2015). PARP inhibitors effectively inhibit the repair of DNA single-strand breaks. In OC cases linked with BRCA mutations or HRD, PARP inhibitors exhibit superior efficacy due to compromised DNA repair mechanisms that culminate in cell demise. Our subgroup analysis based on HR status indicated that the PFS benefit of PARP inhibitors varies, with the advantages diminishing in the following order: germline BRCA mutation (HR = 0.256), BRCA mutation (HR = 0.341), HRD (HR = 0.427), non-germline BRCA mutation (HR = 0.450), BRCA wild-type (HR = 0.523), and HRP (HR = 0.615). This gradient suggests that wider availability and accessibility of tumor HRD testing could be pivotal in guiding therapeutic decisions regarding PARP inhibitor maintenance in OC. Additionally, our subgroup analysis indicated that the OS benefit of PARP maintenance therapy is similar in patients with BRCA mutations (HR = 0.701) and those with germline BRCA mutations (HR = 0.738). Further investigation is necessary to understand the OS benefits of PARP maintenance therapy across different HR statuses, as more comprehensive OS data from future trials become available.

To date, the FDA has approved three PARP inhibitors-olaparib, niraparib, and rucaparib-for clinical use in OC patients. Olaparib, the first PARP inhibitor introduced into clinical practice, has been utilized for both maintenance and treatment of OC, supported by several highly successful clinical trials (Giannini et al., 2023). Study 19 assessed olaparib’s efficacy in the maintenance setting for relapsed, platinum-sensitive OC across all patients, demonstrating significantly longer PFS with olaparib compared to placebo (Ledermann et al., 2012). SOLO-2 specifically targeted high-grade serous OC with BRCA1/2 mutations, revealing that olaparib significantly prolonged PFS relative to placebo (Pujade-Lauraine et al., 2017). Rucaparib, the second approved PARP inhibitor, received accelerated FDA approval as a monotherapy, and subsequently for maintenance treatment (Hirschl et al., 2024). The ARIEL 3 trial, which randomized eligible patients to receive either rucaparib or placebo as maintenance therapy, showed that rucaparib significantly enhanced PFS in patients with platinum-sensitive OC who had responded to platinum-based CT. Notably, rucaparib markedly improved PFS in patients with known genomic or somatic BRCA mutations. For the HRD subgroup, PFS was 13.6 months compared to 5.4 months (HR: 0.32, 95% CI: 0.24–0.42), and in the intention-to-treat population, it was 10.8 months versus 5.4 months (HR: 0.36, 95% CI: 0.30–0.45) (Coleman et al., 2017). A recent meta-analysis confirmed rucaparib’s significant efficacy in enhancing PFS and objective response rate in OC patients, particularly those with BRCA mutation (Mustafa et al., 2024). Additionally, niraparib is the latest PARP inhibitor approved for maintenance treatment in OC. Similar to the SOLO-2 findings for olaparib, the PRIMA trial included patients without deleterious BRCA1/2 mutations and showed a significant PFS benefit with niraparib monotherapy across the overall population, regardless of HRD status (González-Martín et al., 2019). Our meta-analysis, which synthesized data from existing RCTs, confirmed that maintenance therapy with olaparib, niraparib, or rucaparib significantly improves PFS compared to placebo. Additionally, maintenance therapy with olaparib or niraparib was associated with a significant extension in OS in OC patients. Nevertheless, determining the most effective PARP inhibitor among olaparib, niraparib, and rucaparib for OC remains challenging due to the absence of RCTs that directly compare their efficacies. Moreover, a feasibility study comparing PARP inhibitor maintenance therapies for OC indicated that indirect treatment comparisons, such as network meta-analyses and population-adjusted indirect comparisons, should be performed with caution due to confounding factors that can preclude objective systematic comparison across RCTs (Lorusso et al., 2022). Despite this, our subgroup analysis suggests that olaparib may offer superior efficacy in enhancing PFS and OS when indirectly comparing HR values. This conclusion, however, necessitates further validation through rigorously designed future research.

TFST and TSST serve as valuable endpoints in evaluating disease recurrence and the initiation of subsequent treatments, reflecting a prolonged PFS benefit and indicating a potential OS advantage (Matulonis et al., 2015). Furthermore, an extended in CFI suggests that patients on PARP inhibitors can delay additional cancer therapies, giving them more time to recover from the adverse effects of prior CT and defer the side effects of further anticancer treatments (Ledermann et al., 2020). In this meta-analysis, patients receiving PARP inhibitor maintenance therapy demonstrated a significant improvement in CFI, TFST, and TSST compared to those on placebo. Subgroup analyses further revealed that the benefit of PARP inhibitor maintenance therapy on these endpoints was consistent, irrespective of HR status, OC subtypes, or the specific PARP inhibitor used. Similar enhancements in post-progression outcomes have been documented in clinical trials evaluating PARP inhibitors for second-line maintenance in OC. For instance, the NOVA trial revealed that maintenance therapy with niraparib significantly improved median CFI and TFST compared to placebo, both in patients with germline BRCA mutations and those without (Mirza et al., 2016). Likewise, the SOLO-2 trial showed that maintenance olaparib significantly extended median TFST and TSST in patients harboring BRCA mutations relative to placebo (Pujade-Lauraine et al., 2017).

Beyond demonstrating the substantial efficacy of PARP inhibitor maintenance therapy in OC, our study also verified an increased risk of any grade and grade ≥3 TEAEs. This elevated risk was consistently observed in all subgroup analyses. Previous investigations have identified fatigue, nausea, anemia, neutropenia, and thrombocytopenia as prevalent grade ≥3 AEs associated with PARP inhibitor therapy (Banerjee et al., 2021; Coleman et al., 2019; DiSilvestro et al., 2023; González-Martín et al., 2019; Li et al., 2022b; Ray-Coquard et al., 2019). Furthermore, a recent meta-analysis has corroborated that PARP inhibitors are linked with a distinct toxicity profile, predominantly involving hematological abnormalities, with a higher incidence of anemia, thrombocytopenia, and neutropenia compared to placebo (Zhou et al., 2024). Another meta-analysis on safety profiles also reported that the most frequent AEs included fatigue, nausea, vomiting, anemia, and neutropenia, a finding supported by the majority of reviewed studies (Baradács et al., 2024). Thus, it is needed for clinicians to continuously monitor OC patients undergoing PARP inhibitor maintenance treatment, ensuring timely identification and management of TEAEs to mitigate potential health risks.

Nonetheless, this research is not without its limitations. First, this analysis was conducted using aggregate study-level data rather than individual patient data. We did not present separate data for the use of PARP inhibitors in initial and recurrent treatments; however, this form of analysis has already been conducted in previously published meta-analysis (Ruscito et al., 2020). Second, the observed heterogeneity in PFS across studies may stem from various factors, including the stage of OC, types of PARP inhibitors, follow-up duration, and the diverse ethnic backgrounds of participants. Third, while the efficacy of PARP inhibitors is well established in population with HRD and BRCA mutations (Shao et al., 2021), further research is needed to explore their role in HRP population. Fourth, OC is predominantly diagnosed in older adults, who constitute the majority of cases observed in clinical settings (Masvidal Hernandez et al., 2024). The insufficient number of included RCTs that provide HRs and 95% CIs for efficacy and safety outcomes across various age groups restricts our ability to perform further age-based subgroup analyses. Furthermore, future research should focus on assessing the effects of PARP inhibitors on quality of life, as the influence of these maintenance therapies on the quality of life of OC patients remains unreported (Masvidal Hernandez et al., 2024). Fifth, prior research has highlighted that the selection of maintenance therapy should be informed by several key considerations: (1) molecular biomarkers, including BRCA1/2 mutations and HRD status; (2) disease-specific factors, such as chemotherapy response score, the stage at diagnosis, and residual disease post-surgery; and (3) patient characteristics, encompassing comorbidities and concurrent medications (Perez-Fidalgo et al., 2024). While our study has considered BRCA1/2 and HRD status, additional subgroup analyses should be conducted based on these other variables. Finally, although olaparib and niraparib have been extensively studied, fuzuloparib and senaparib have only been investigated in a single trial. Additional studies are needed to confirm the efficacy and safety of fuzuloparib and senaparib in women with OC.

## 5 Conclusion

In conclusion, the findings from this meta-analysis demonstrated that PARP inhibitors play a significant role in maintenance therapy for OC, showing improvements in PFS, OS, CFI, TFST, and TSST. Subgroup analysis further revealed that this maintenance therapy markedly improved PFS compared to placebo, irrespective of HR status. Nevertheless, the use of PARP inhibitors for maintenance was associated with a heightened risk of any grade and grade ≥3 TEAEs. It is crucial for clinicians to monitor and manage TEAEs when utilizing PARP inhibitors for maintenance therapy in OC within clinical practice.

## Data Availability

The original contributions presented in the study are included in the article/Supplementary Material, further inquiries can be directed to the corresponding author.
