# Supplementary material for: Efficacy and safety of PARP inhibitor maintenance therapy for ovarian cancer: a meta-analysis and trial sequential analysis of randomized controlled trials
Source: Front Pharmacol. 2024 Sep 18;15:1460285. doi: 10.3389/fphar.2024.1460285 (PMC11457084; doi:10.3389/fphar.2024.1460285)

**FIGURE S2** Subgroup analysis based on homologous recombination status for progression-free survival. (A) Subgroup = Homologous recombination deficiency; (B) Subgroup = BRCA mutation; (C) Subgroup = Germline BRCA mutation; (D) Subgroup = Non-germline BRCA mutation; (E) Subgroup = BRCA wild-type; (E) Subgroup = Homologous recombination proficiency.


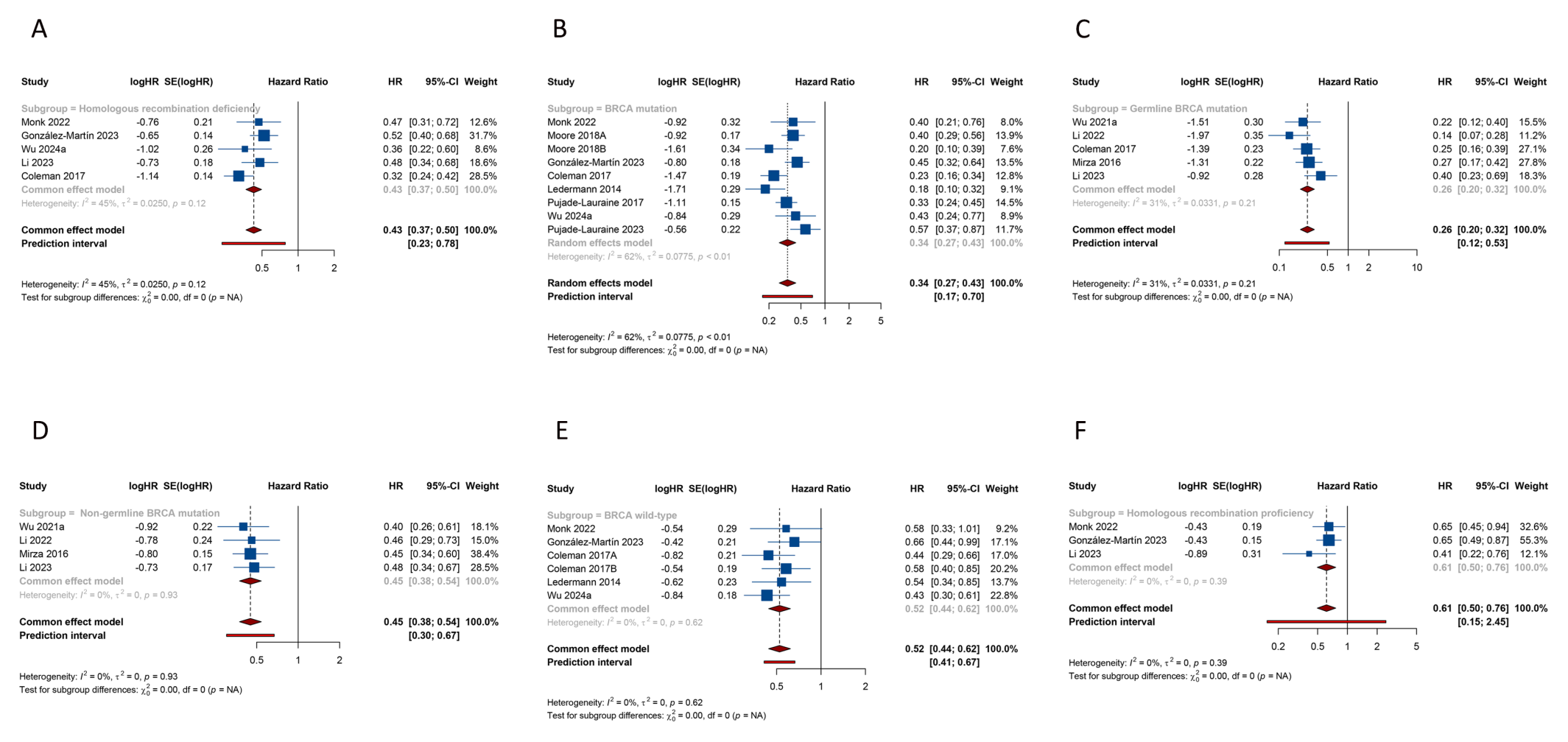


**FIGURE S3** Subgroup analysis based on subtypes of ovarian cancer (OC) for progression-free survival. (A) Subgroup = Newly diagnosed OC; (B) Subgroup = Recurrent OC.


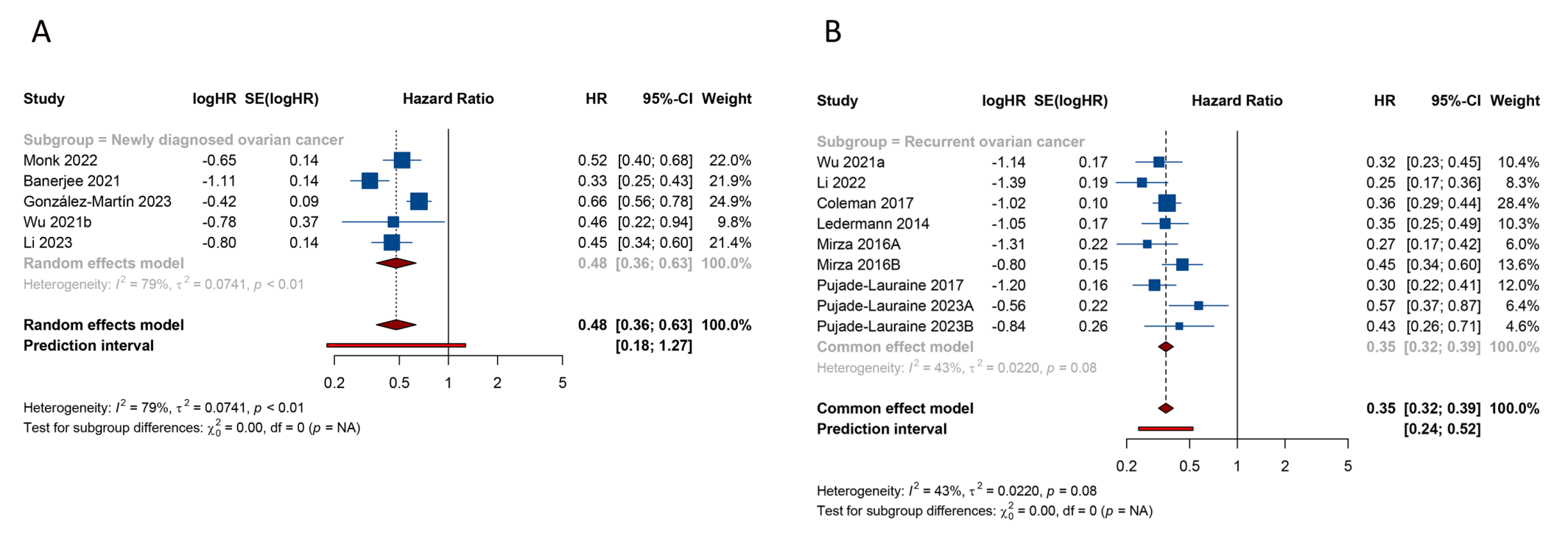


**FIGURE S4** Subgroup analysis based on types of PARP inhibitors for progression-free survival. (A) Subgroup = Olaparib vs. Placebo; (B) Subgroup = Niraparib vs. Placebo; (C) Subgroup = Rucaparib vs. Placebo.


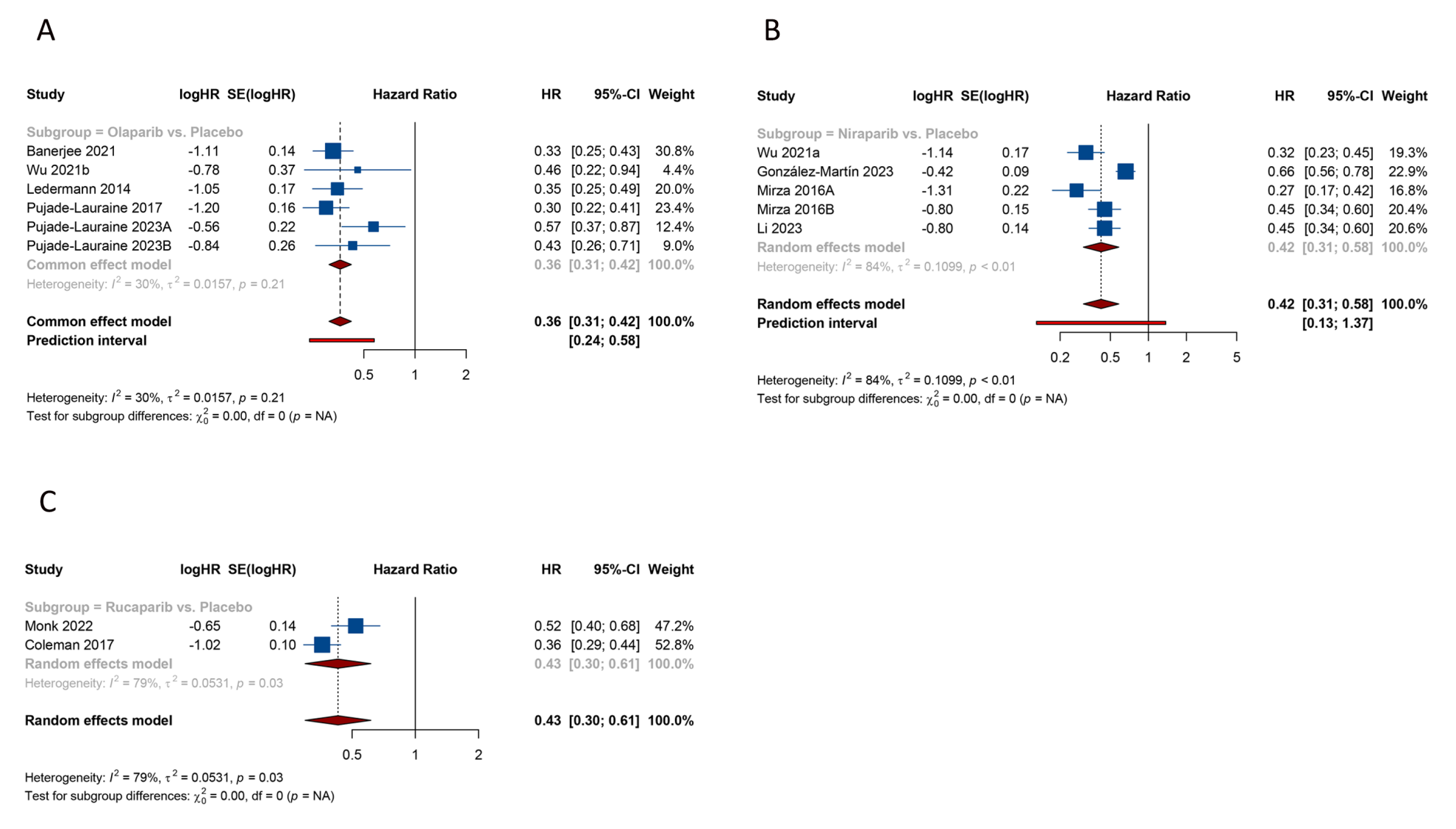


**FIGURE S5** Subgroup analysis based on homologous recombination status for overall survival. (A) Subgroup = Homologous recombination deficiency; (B) Subgroup = BRCA mutation; (C) Subgroup = Germline BRCA mutation.


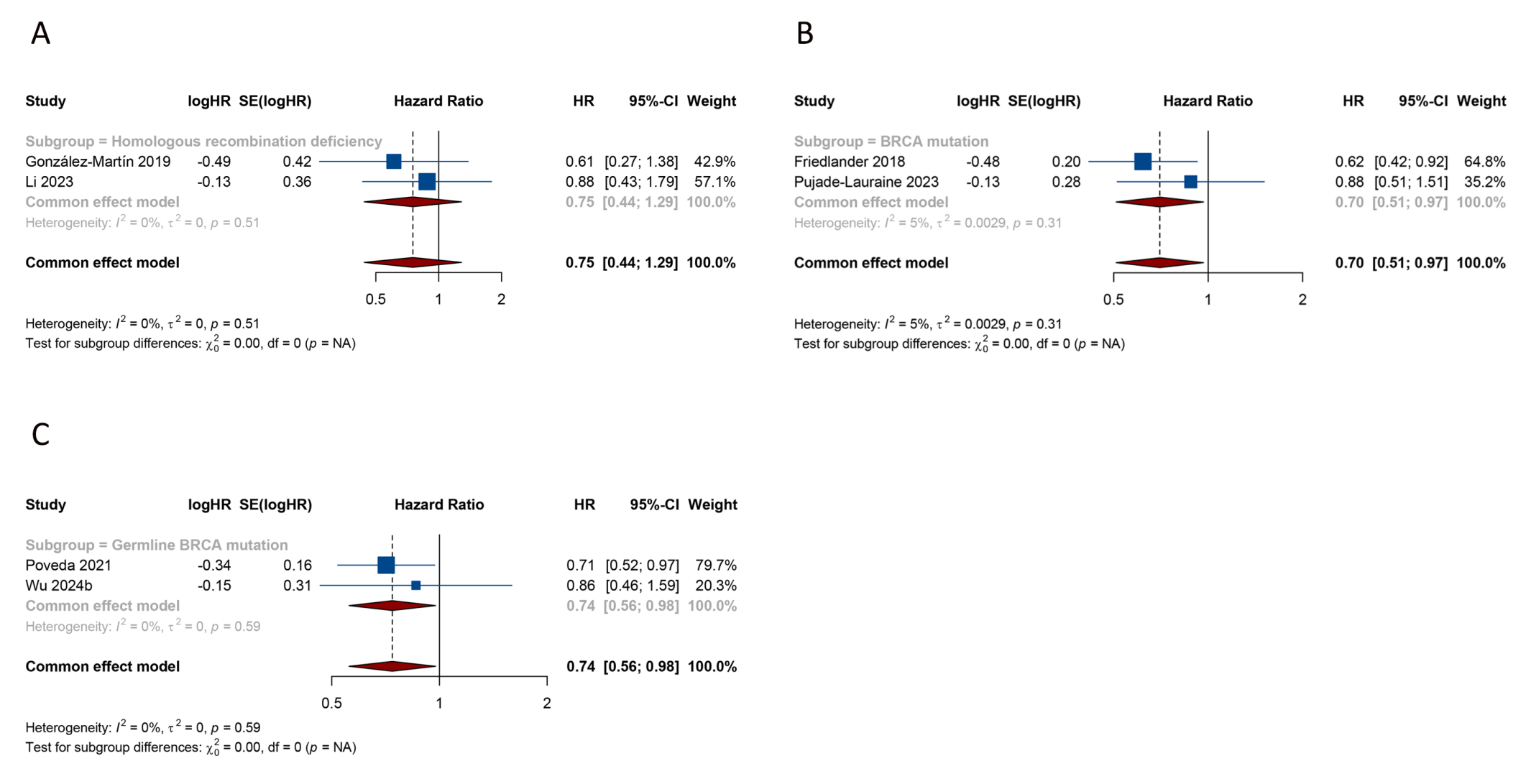


**FIGURE S6** Subgroup analysis based on subtypes of ovarian cancer (OC) for overall survival. (A) Subgroup = Newly diagnosed OC; (B) Subgroup = Recurrent OC.


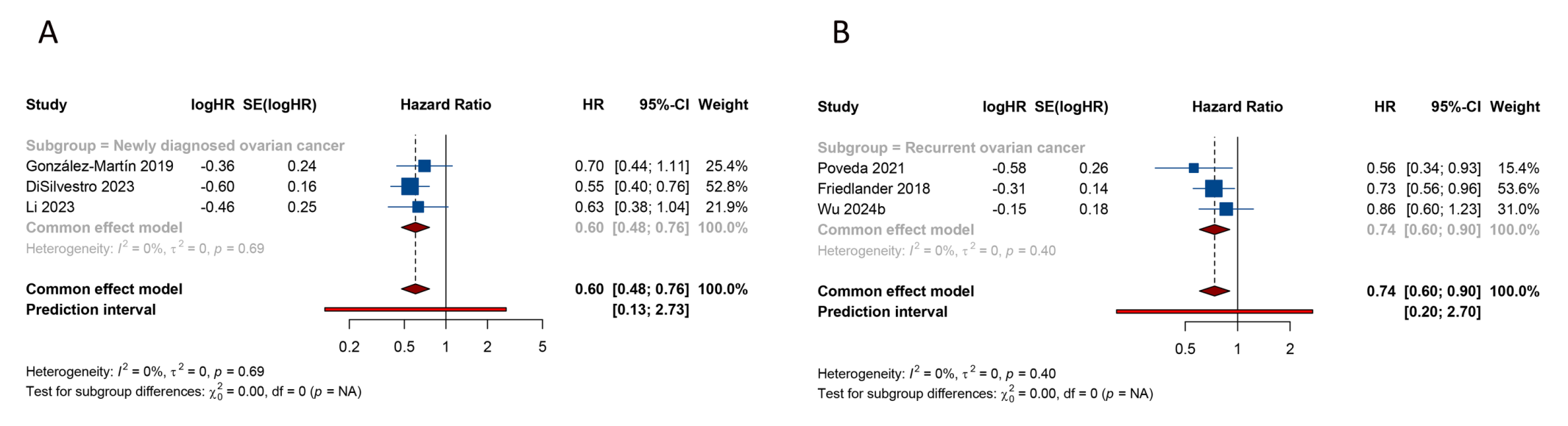


**FIGURE S7** Subgroup analysis based on types of PARP inhibitors for overall survival. (A) Subgroup = Olaparib vs. Placebo; (B) Subgroup = Niraparib vs. Placebo.


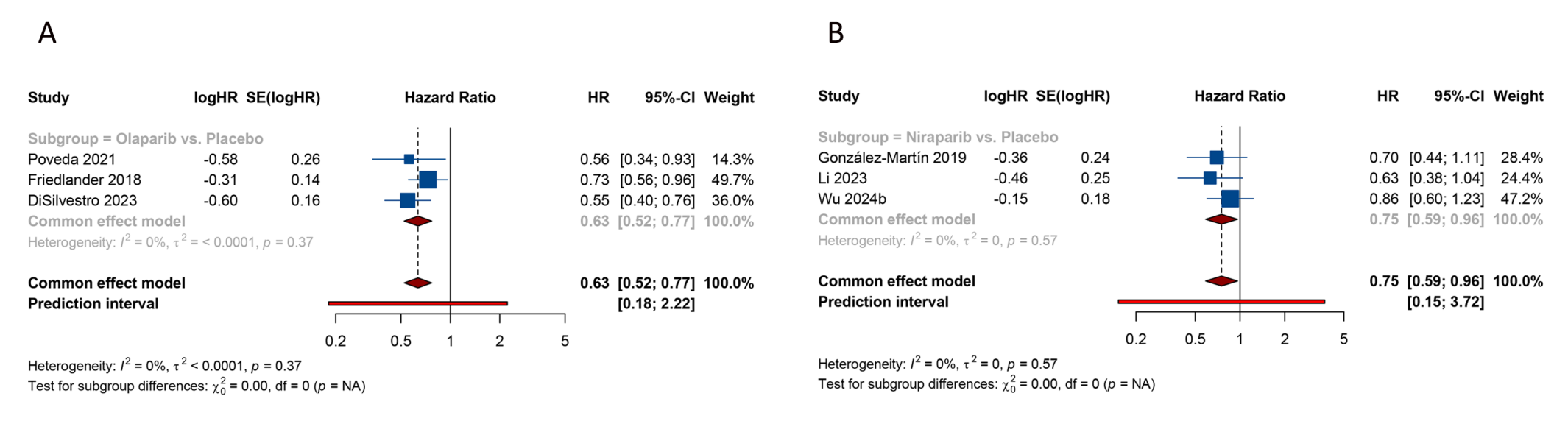


**FIGURE S8** Subgroup analysis based on subtypes of ovarian cancer (OC) or types of PARP inhibitors for chemotherapy-free interval. (A) Subgroup = Recurrent OC; (B) Subgroup = Niraparib vs. Placebo.


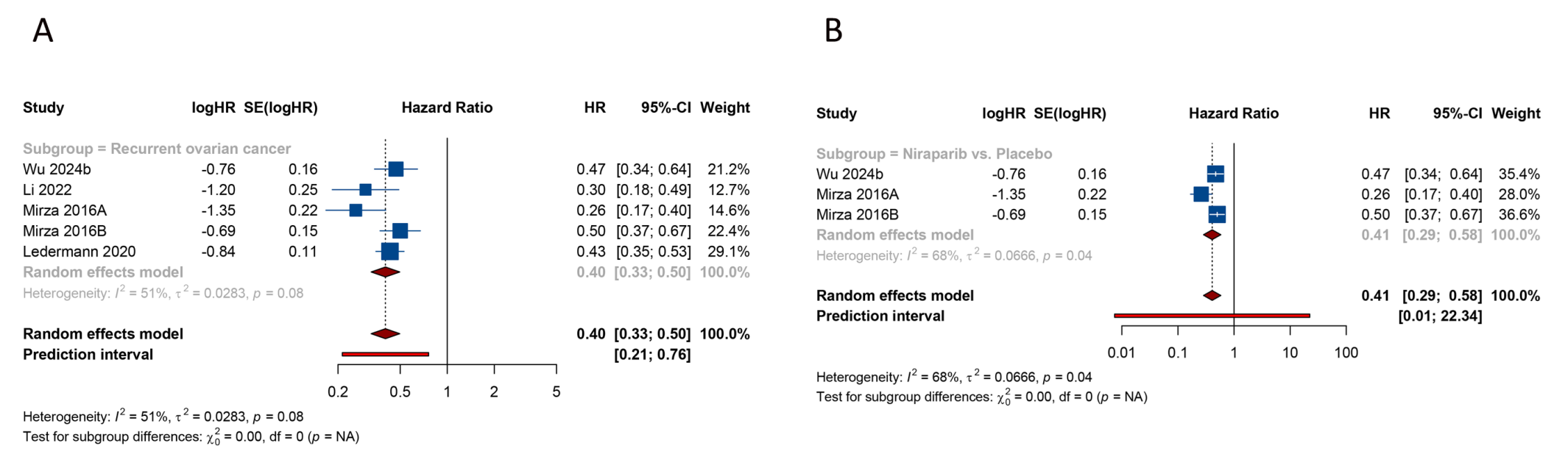


**FIGURE S9** Subgroup analysis based on homologous recombination status for time to first subsequent therapy or death. (A) Subgroup = Homologous recombination deficiency; (B) Subgroup = BRCA mutation.


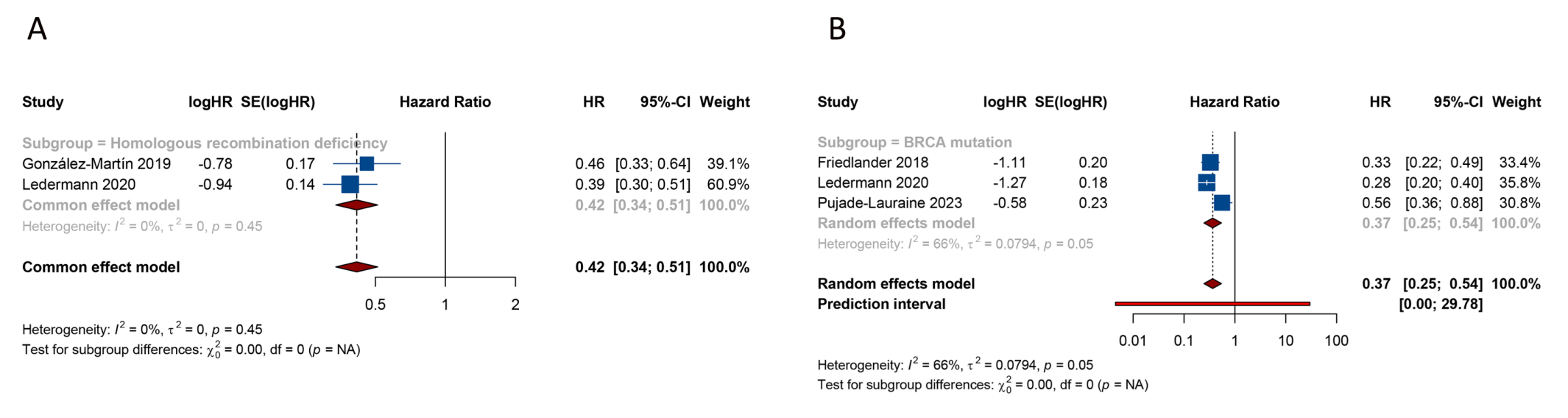


**FIGURE S10** Subgroup analysis based on subtypes of ovarian cancer (OC) for time to first subsequent therapy or death. (A) Subgroup = Newly diagnosed OC; (B) Subgroup = Recurrent OC.


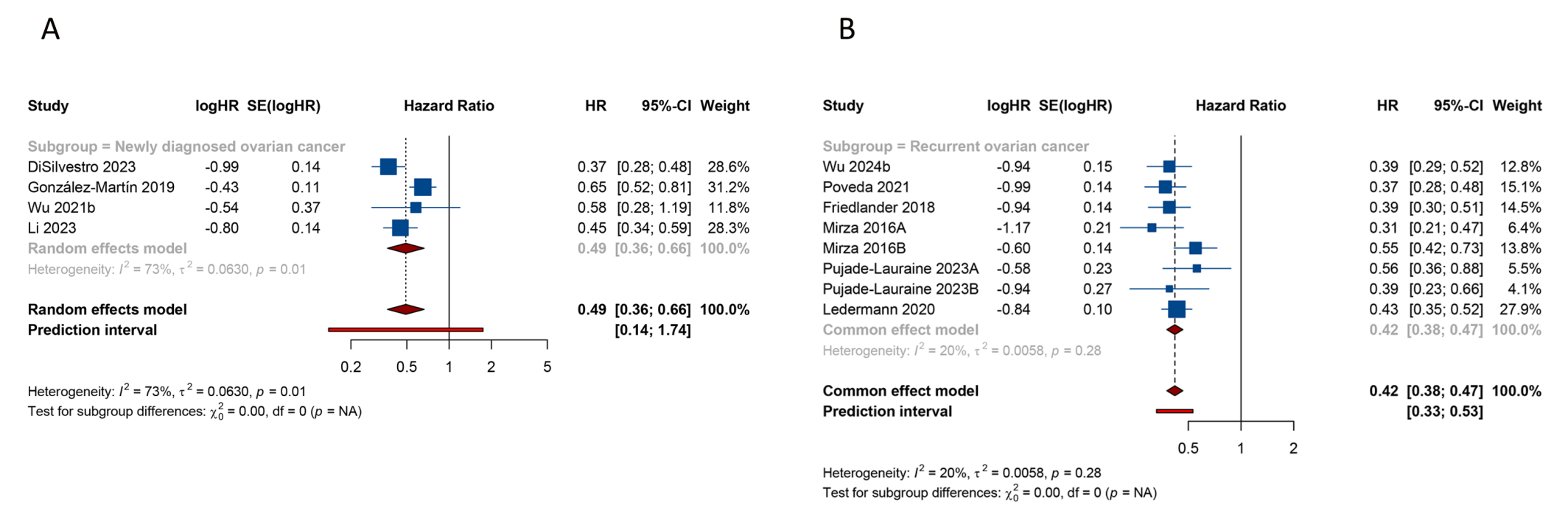


**FIGURE S11** Subgroup analysis based on types of PARP inhibitors for time to first subsequent therapy or death. (A) Subgroup = Olaparib vs. Placebo; (B) Subgroup = Niraparib vs. Placebo.


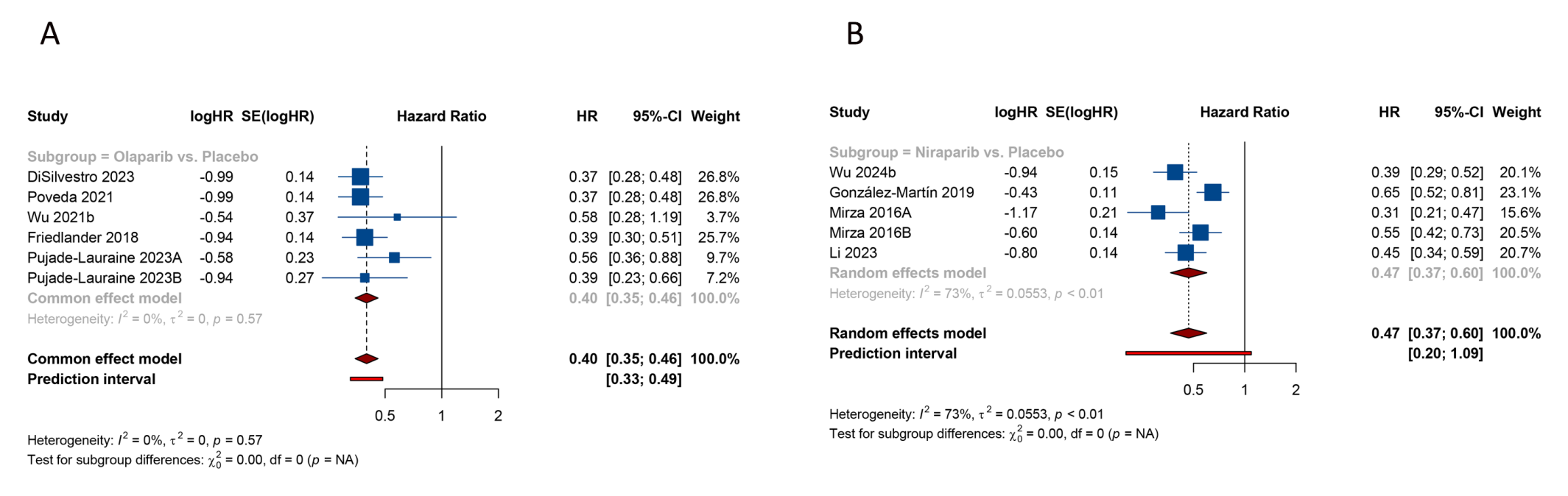


**FIGURE S12** Subgroup analysis based on homologous recombination status, subtypes of ovarian cancer (OC), or types of PARP inhibitors for time to second subsequent therapy or death. (A) Subgroup = BRCA mutation; (B) Subgroup = Newly diagnosed OC; (C) Recurrent OC; (D) Olaparib vs. Placebo.


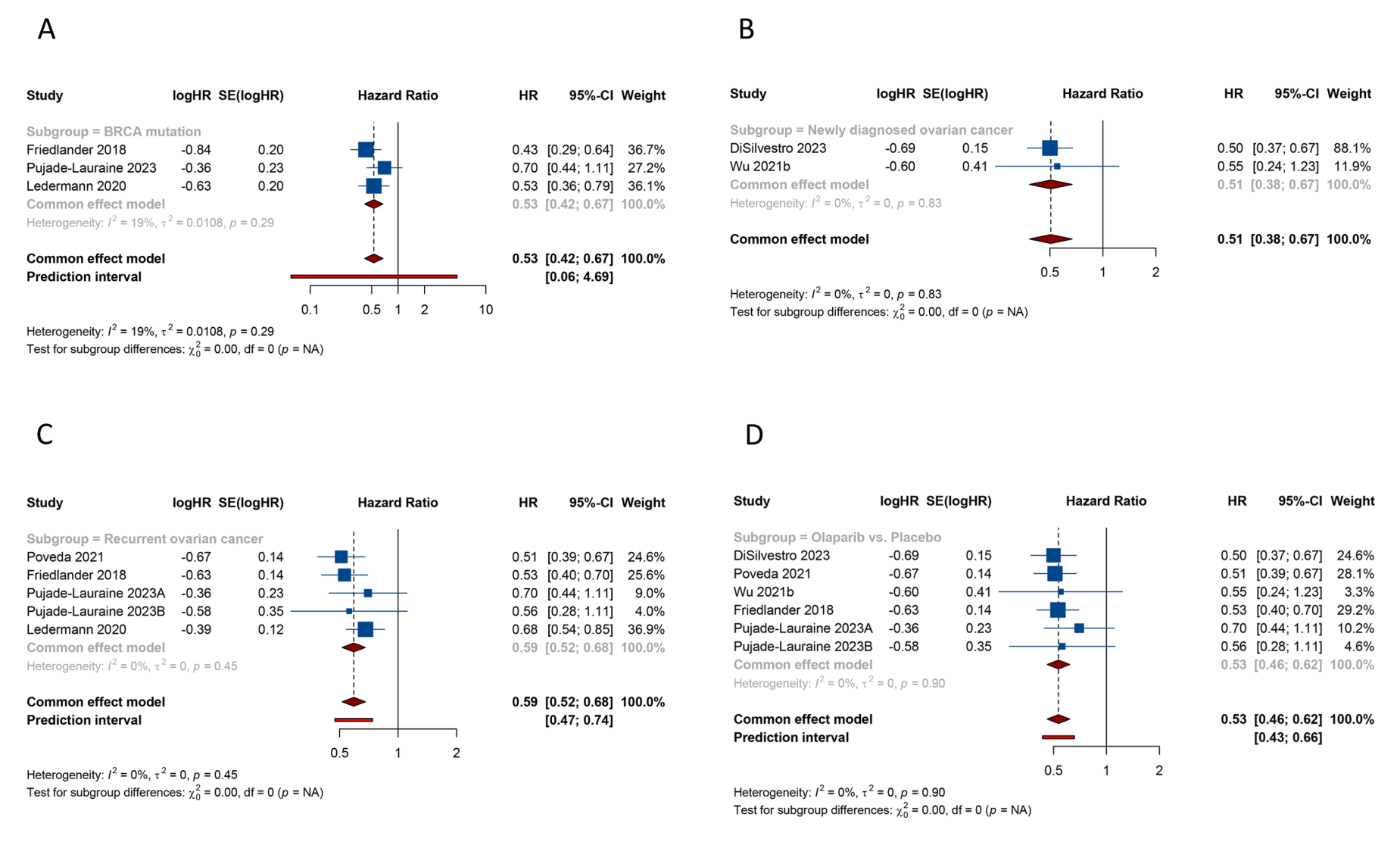


**FIGURE S13** Subgroup analysis based on subtypes of ovarian cancer (OC) for treatment-emergent adverse events of any grade. (A) Subgroup = Newly diagnosed OC; (B) Subgroup = Recurrent OC.


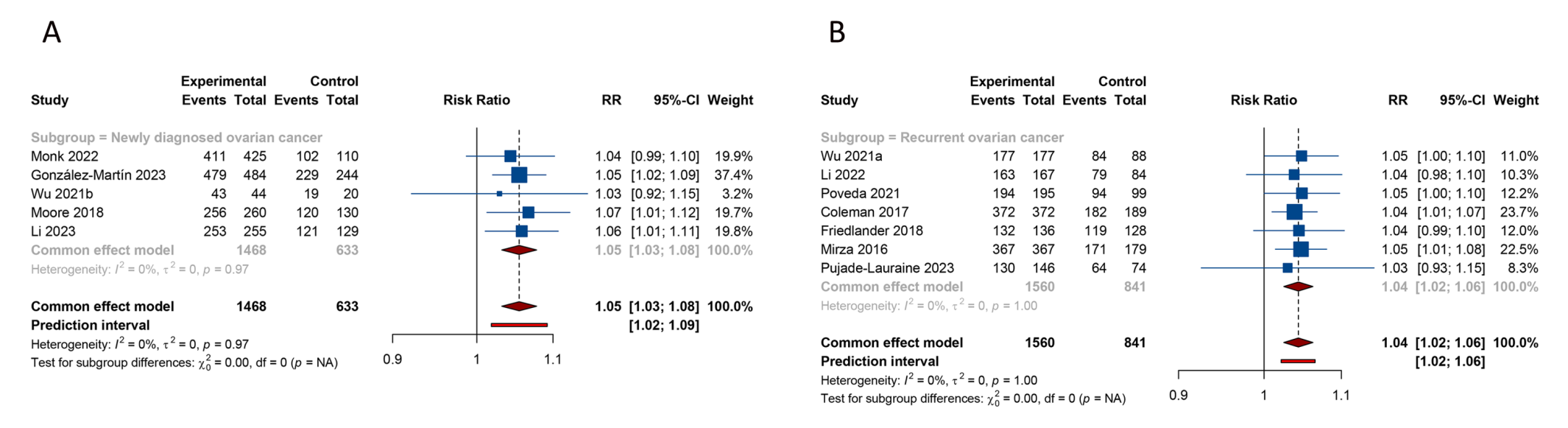


**FIGURE S14** Subgroup analysis based on types of PARP inhibitors for treatment-emergent adverse events of any grade. (A) Subgroup = Olaparib vs. Placebo; (B) Subgroup = Niraparib vs. Placebo; (C) Subgroup = Rucaparib vs. Placebo.


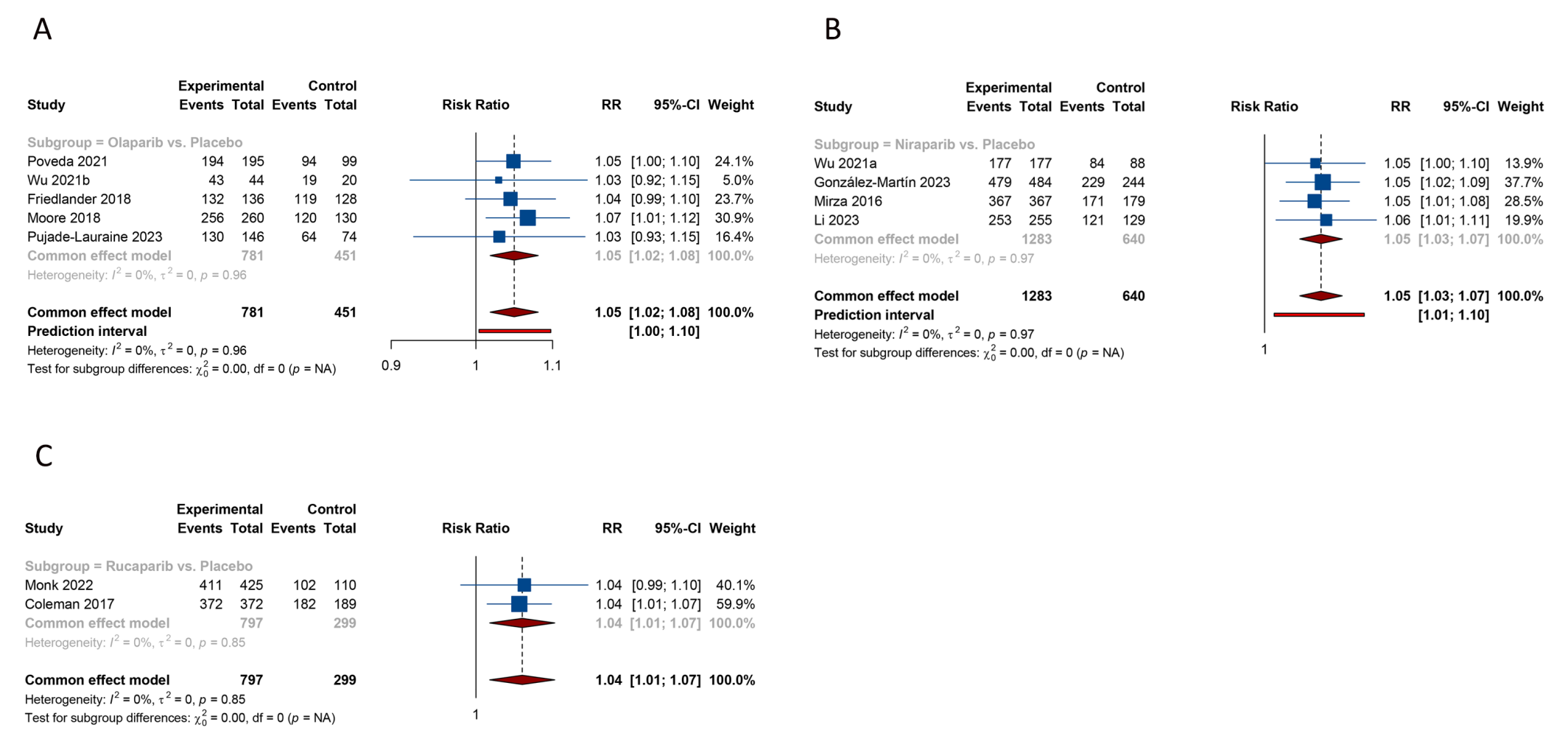


**FIGURE S15** Subgroup analysis based on subtypes of ovarian cancer (OC) for grade ≥ 3 treatment-emergent adverse events. (A) Subgroup = Newly diagnosed OC; (B) Subgroup = Recurrent OC.


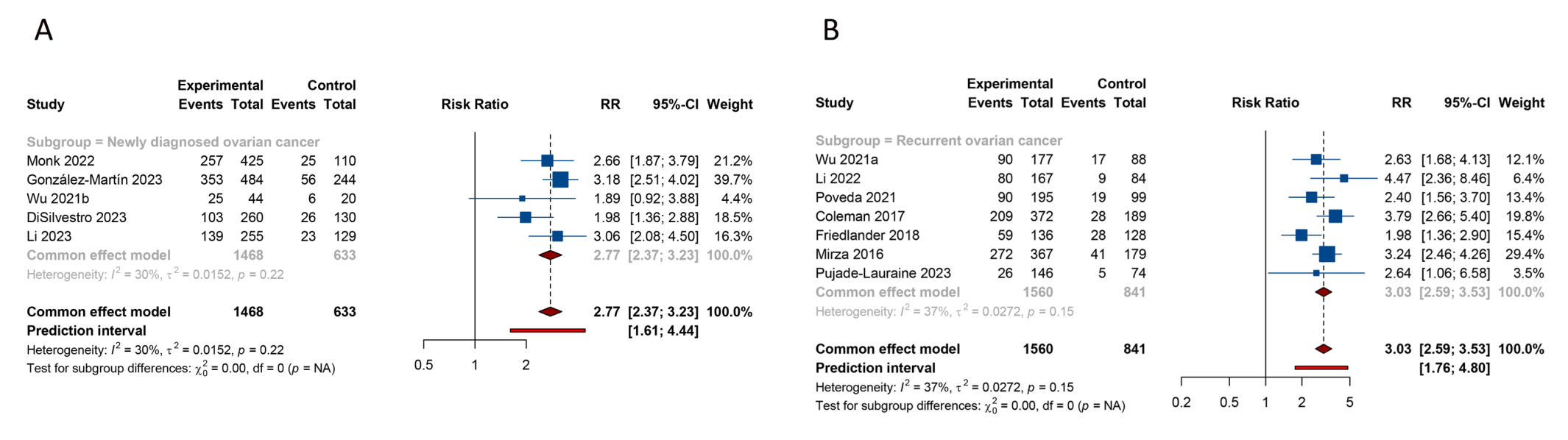


**FIGURE S16** Subgroup analysis based on types of PARP inhibitors for grade ≥ 3 treatment-emergent adverse events. (A) Subgroup = Olaparib vs. Placebo; (B) Subgroup = Niraparib vs. Placebo; (C) Subgroup = Rucaparib vs. Placebo.


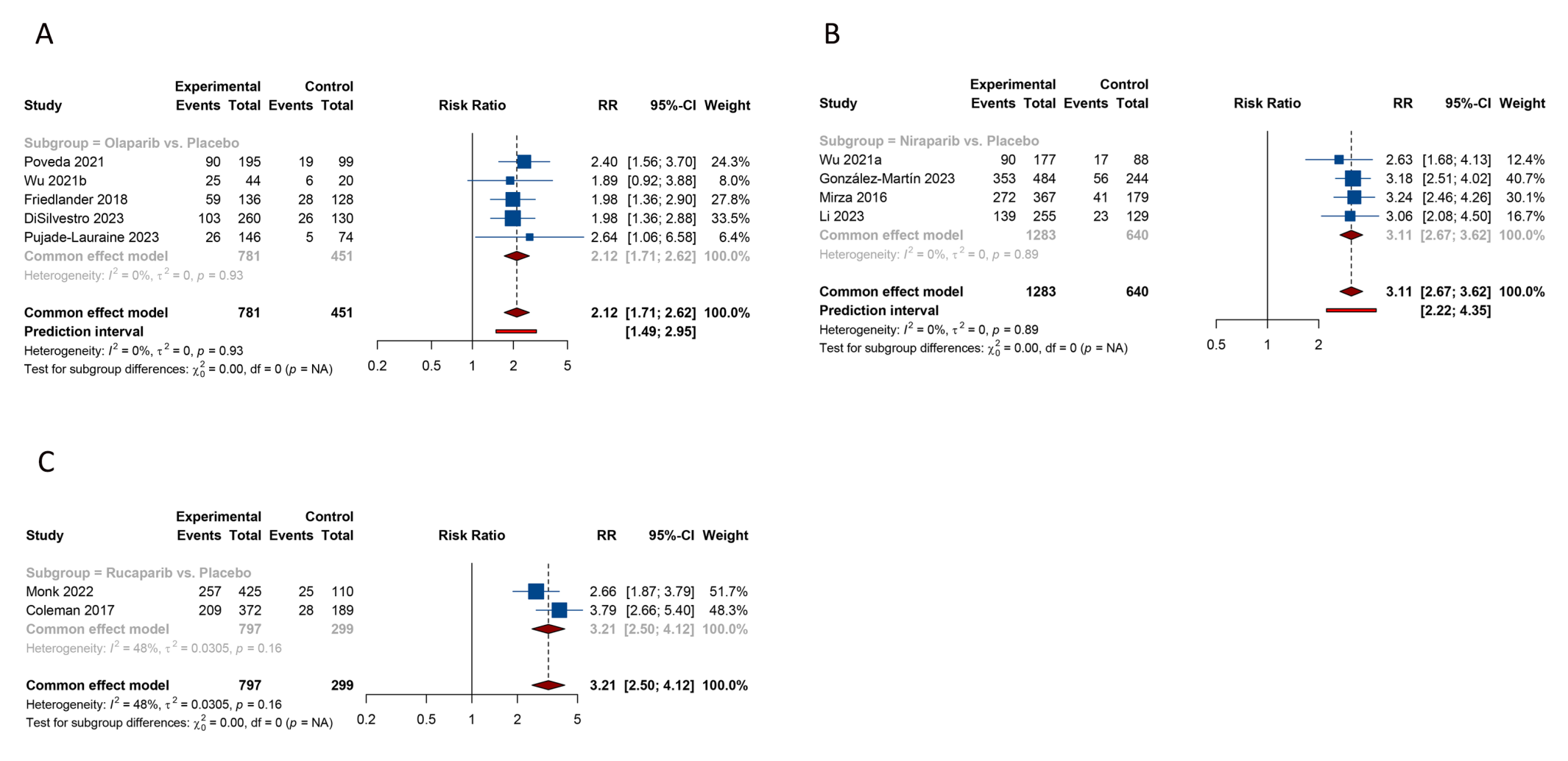


**FIGURE S17** Sensitivity analysis of progression-free survival (A) and overall survival (B) after PARP inhibitor maintenance therapy for ovarian cancer.


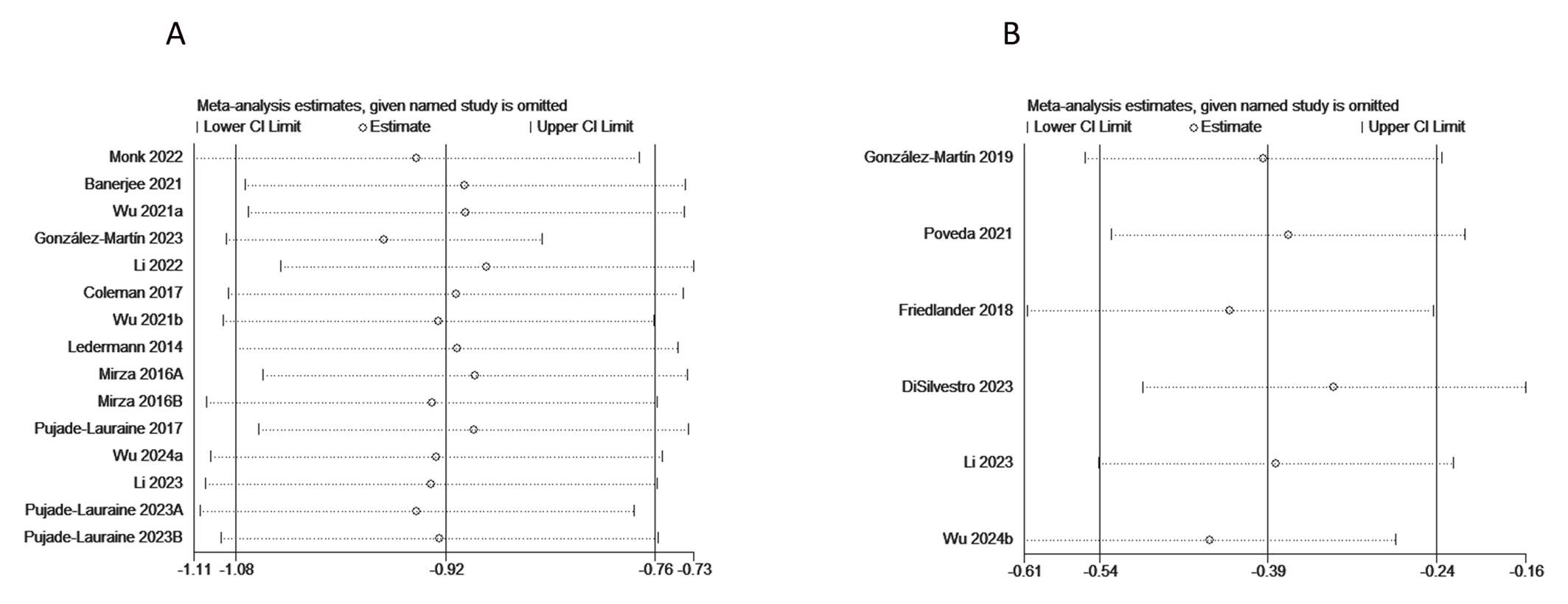


**FIGURE S18** Sensitivity analysis of secondary outcomes after PARP inhibitor maintenance therapy for ovarian cancer. (A) Chemotherapy-free interval; (B) Time to first subsequent therapy or death; (C) Time to second subsequent therapy or death; (D) Any grade treatment-emergent adverse events (TEAEs); (E) Grade ≥ 3 TEAEs.


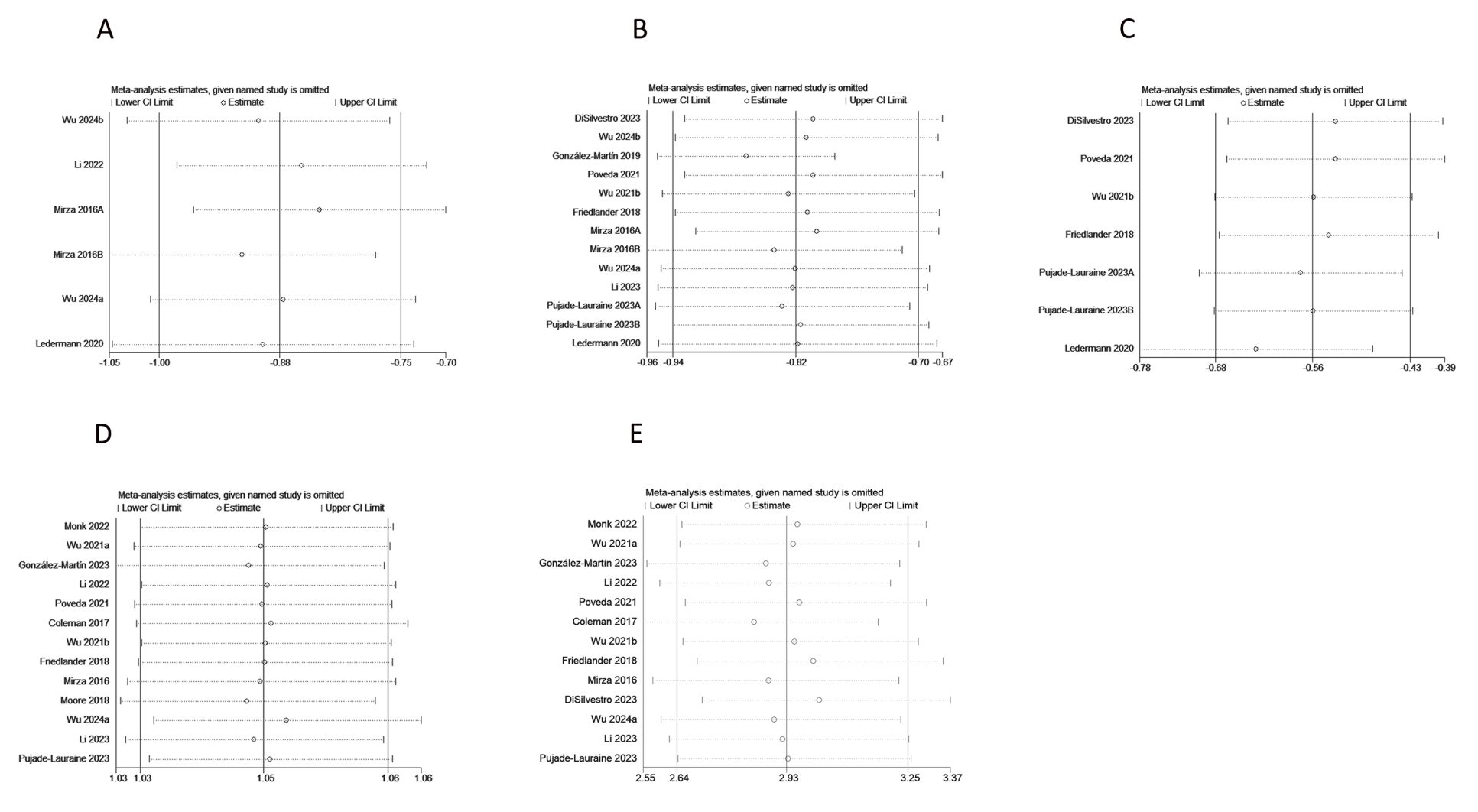


**FIGURE S19** Funnel plot of progression-free survival (A) and overall survival (B) after PARP inhibitor maintenance therapy for ovarian cancer.


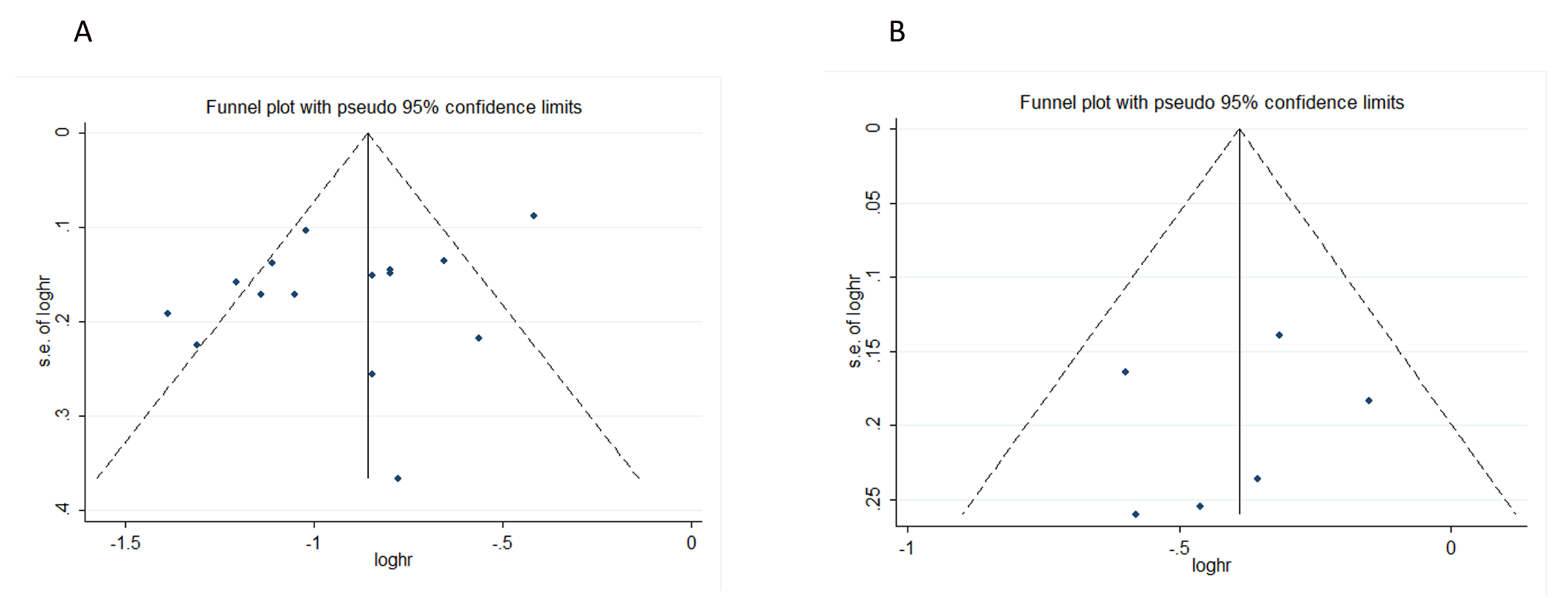


**FIGURE S20** Funnel plot of secondary outcomes after PARP inhibitor maintenance therapy for ovarian cancer. (A) Chemotherapy-free interval; (B) Time to first subsequent therapy or death; (C) Time to second subsequent therapy or death; (D) Any grade treatment-emergent adverse events (TEAEs); (E) Grade ≥ 3 TEAEs.


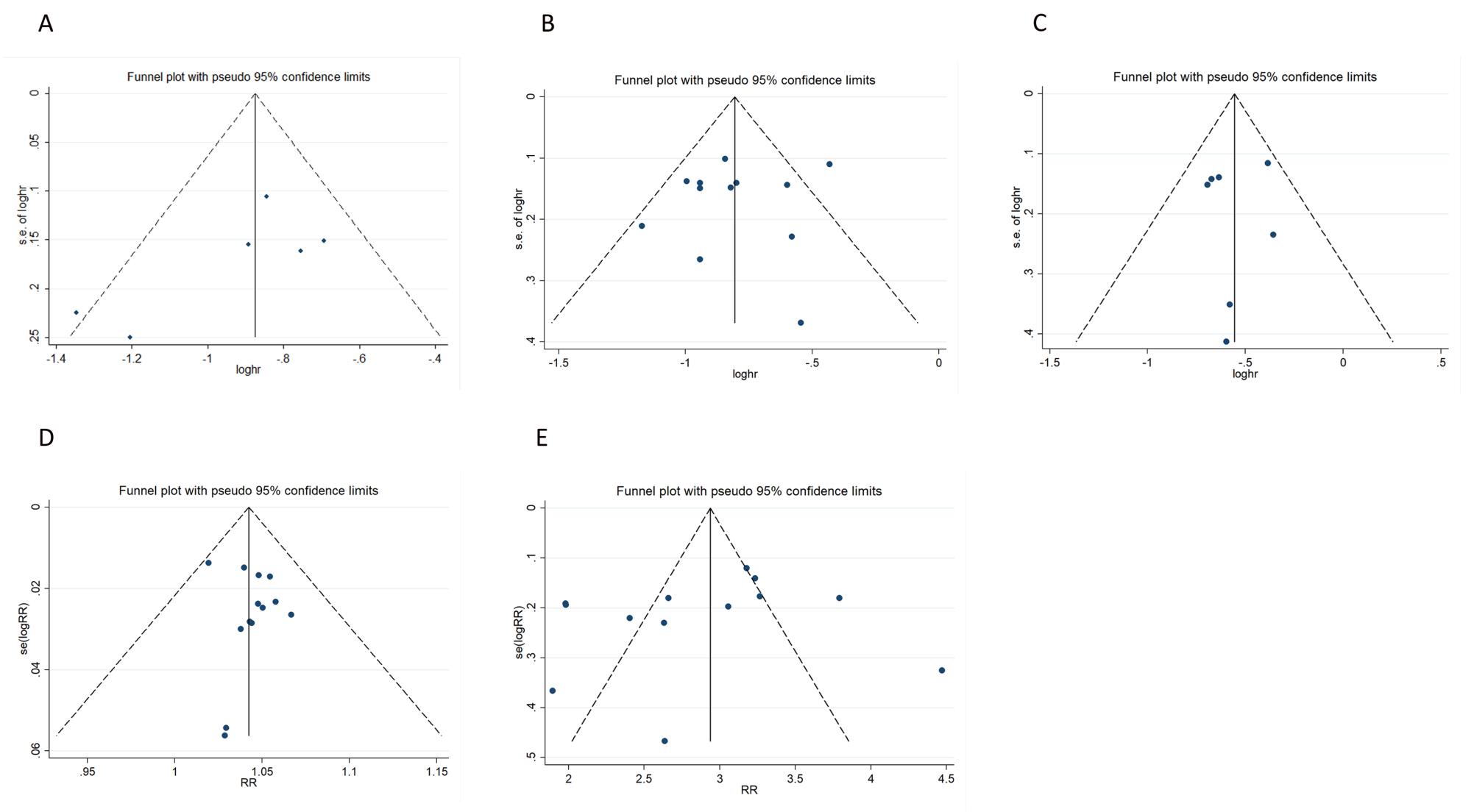

Supplement: Supplementary file 1 [file DataSheet4.DOCX]
