# Supplementary material for: Efficacy and safety of PARP inhibitor maintenance therapy for ovarian cancer: a meta-analysis and trial sequential analysis of randomized controlled trials
Source: Front Pharmacol. 2024 Sep 18;15:1460285. doi: 10.3389/fphar.2024.1460285 (PMC11457084; doi:10.3389/fphar.2024.1460285)
Supplement: Supplementary file 2 [file DataSheet3.DOCX]

| TABLE S1 Quality analysis of the included RCTs by modified Jadad scale. | | | | | | |
| --- | --- | --- | --- | --- | --- | --- |
| Study | Randomization | Randomization concealment | Double blind | Withdrawals and dropouts | Score | Study quality |
| Monk (2022) | 2 | 2 | 2 | 1 | 7 | High |
| Banerjee (2021) | 2 | 2 | 2 | 1 | 7 | High |
| Wu (2021a) | 2 | 2 | 2 | 1 | 7 | High |
| González-Martín (2019) | 2 | 2 | 2 | 1 | 7 | High |
| Li (2022) | 2 | 2 | 2 | 1 | 7 | High |
| Poveda (2021) | 2 | 2 | 2 | 1 | 7 | High |
| Coleman (2017) | 2 | 2 | 2 | 1 | 7 | High |
| Wu (2021b) | 1 | 1 | 2 | 1 | 5 | High |
| Friedlander (2018) | 2 | 2 | 2 | 1 | 7 | High |
| Ledermann (2014) | 2 | 2 | 2 | 1 | 7 | High |
| Mirza (2016) | 2 | 2 | 2 | 1 | 7 | High |
| Moore (2018) | 2 | 2 | 2 | 1 | 7 | High |
| Pujade-Lauraine (2017) | 2 | 2 | 2 | 1 | 7 | High |
| Wu (2024) | 2 | 2 | 2 | 1 | 7 | High |
| Li (2023) | 2 | 2 | 2 | 1 | 7 | High |
| González-Martín (2023) | 2 | 2 | 2 | 1 | 7 | High |
| DiSilvestro (2023) | 2 | 2 | 2 | 1 | 7 | High |
| Wu (2024b) | 2 | 2 | 2 | 1 | 7 | High |
| Pujade-Lauraine (2023) | 2 | 2 | 2 | 1 | 7 | High |
| Ledermann (2020) | 2 | 2 | 2 | 1 | 7 | High |


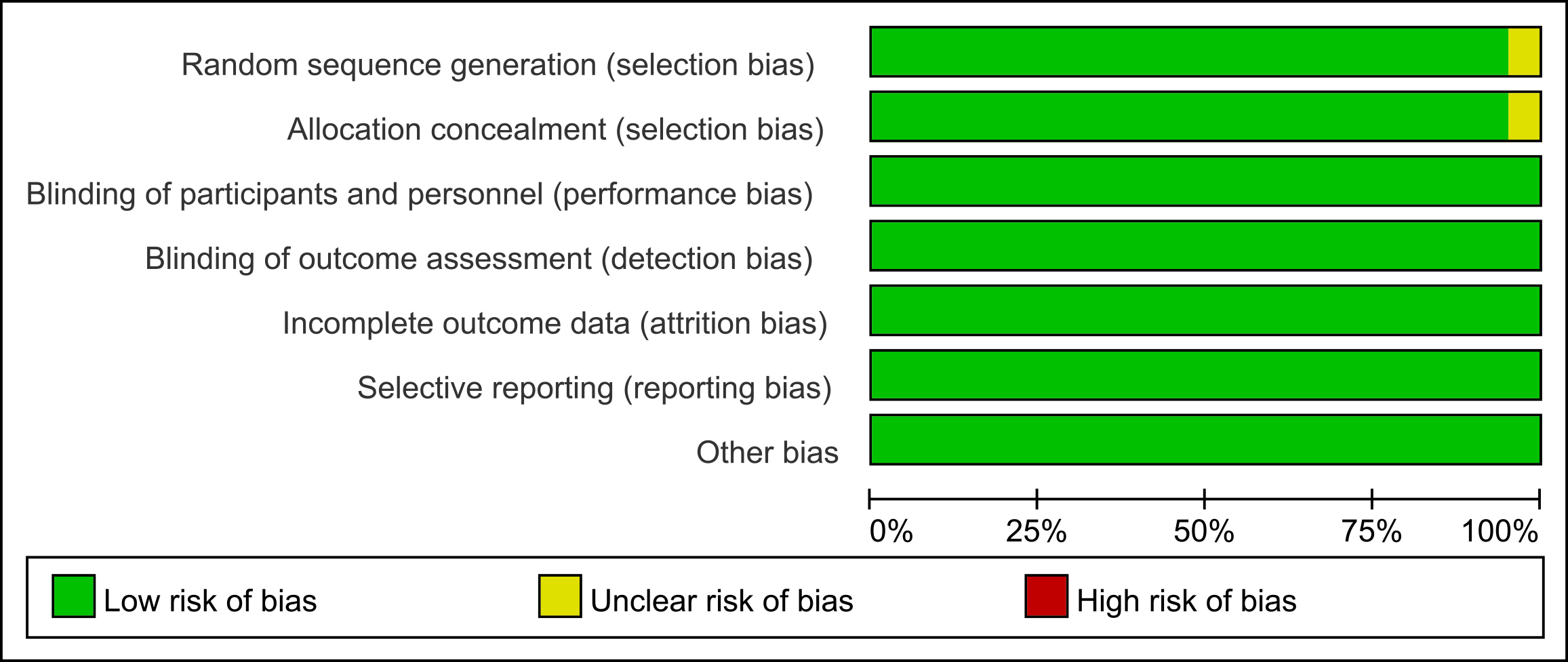


**FIGURE S1** Risk of Bias graph of the included randomized controlled trials.
