# Supplementary material for: Efficacy and safety of PARP inhibitor maintenance therapy for ovarian cancer: a meta-analysis and trial sequential analysis of randomized controlled trials
Source: Front Pharmacol. 2024 Sep 18;15:1460285. doi: 10.3389/fphar.2024.1460285 (PMC11457084; doi:10.3389/fphar.2024.1460285)
Supplement: Supplementary file 3 [file DataSheet2.DOCX]

**PubMed 363**

#1 (poly(ADP-ribose) polymerase inhibitor) OR (PARP inhibitor) OR (PARPi) OR (PARP inhibitors) OR (olaparib) OR (lynparza) OR (rucaparib) OR (talazoparib) OR (niraparib) OR (veliparib) OR (rubraca) OR (talzenna) OR (zejula) OR (AZD 2281) OR (AZD221) OR (ABT 888) OR (MK 4827) OR (AG014699) OR (BMN 673) OR (PF-01367338)

#2 ((ovar*) AND (cancer* OR neoplas* OR tumor* OR tumour* OR carcinoma* OR adenocarcinoma* OR malignan*)) OR (cancer of ovary) OR (cancer of the ovary)

#3 (randomized controlled trial) OR (RCT) OR (controlled clinical trial) OR (random*)

#4 #1 AND #2 AND #3

**Web of Science 865**

#1 TS=((poly(ADP-ribose) polymerase inhibitor) OR (PARP inhibitor) OR (PARPi) OR (PARP inhibitors) OR (olaparib) OR (lynparza) OR (rucaparib) OR (talazoparib) OR (niraparib) OR (veliparib) OR (rubraca) OR (talzenna) OR (zejula) OR (AZD 2281) OR (AZD221) OR (ABT 888) OR (MK 4827) OR (AG014699) OR (BMN 673) OR (PF-01367338))

#2 TS=(((ovar*) AND (cancer* OR neoplas* OR tumor* OR tumour* OR carcinoma* OR adenocarcinoma* OR malignan*)) OR (cancer of ovary) OR (cancer of the ovary))

#3 TS=((randomized controlled trial) OR (RCT) OR (controlled clinical trial) OR (random*))

#4 #1 AND #2 AND #3

**Embase 1351**

#1 poly AND 'adp ribose'/exp AND 'polymerase inhibitor'/exp OR 'parp inhibitor'/exp OR parpi OR 'parp inhibitors' OR 'olaparib'/exp OR 'lynparza'/exp OR 'rucaparib'/exp OR 'talazoparib'/exp OR 'niraparib'/exp OR 'veliparib'/exp OR 'rubraca'/exp OR 'talzenna'/exp OR 'zejula'/exp OR 'azd 2281'/exp OR azd221 OR 'abt 888'/exp OR 'mk 4827'/exp OR 'ag014699'/exp OR 'bmn 673'/exp OR 'pf 01367338'/exp

#2 ovar* AND (cancer* OR neoplas* OR tumor* OR tumour* OR carcinoma* OR adenocarcinoma* OR malignan*) OR 'cancer of ovary' OR 'cancer of the ovary'

#3 'randomized controlled trial'/exp OR rct OR 'controlled clinical trial'/exp OR random*

#4 #1 AND #2 AND #3

**The Cochrane Library 875**

#1 All Text=((poly(ADP-ribose) polymerase inhibitor) OR (PARP inhibitor) OR (PARPi) OR (PARP inhibitors) OR (olaparib) OR (lynparza) OR (rucaparib) OR (talazoparib) OR (niraparib) OR (veliparib) OR (rubraca) OR (talzenna) OR (zejula) OR (AZD 2281) OR (AZD221) OR (ABT 888) OR (MK 4827) OR (AG014699) OR (BMN 673) OR (PF-01367338))

#2 All Text=(((ovar*) AND (cancer* OR neoplas* OR tumor* OR tumour* OR carcinoma* OR adenocarcinoma* OR malignan*)) OR (cancer of ovary) OR (cancer of the ovary))

#3 All Text=((randomized controlled trial) OR (RCT) OR (controlled clinical trial) OR (random*))

#4 #1 AND #2 AND #3
